# A drug prescription recommendation system based on novel DIAKID ontology and extensive semantic rules

**DOI:** 10.1007/s13755-024-00286-7

**Published:** 2024-03-23

**Authors:** Kadime Göğebakan, Ramazan Ulu, Rahib Abiyev, Melike Şah

**Affiliations:** 1 Directorate of Information Technologies, Istanbul Technical University, North Cyprus via Mersin 10, Famagusta, Turkey; 2 Department of Nephrology, School of Medicine, Adiyaman University, Adiyaman, Turkey; 3 Computer Engineering Department, Near East University, North Cyprus via Mersin 10, Nicosia, Turkey; 4 Computer Engineering Department, Cyprus International University, North Cyprus via Mersin 10, Nicosia, Turkey

**Keywords:** Ontology, SWRL, Chronic kidney disease, eGFR, Medicine, Type 2 Diabetes Mellitus, Electronic health records, Drug doses, DDIs, K-raising

## Abstract

According to the World Health Organization (WHO) data from 2000 to 2019, the number of people living with Diabetes Mellitus and Chronic Kidney Disease (CKD) is increasing rapidly. It is observed that Diabetes Mellitus increased by 70% and ranked in the top 10 among all causes of death, while the rate of those who died from CKD increased by 63% and rose from the 13th place to the 10th place. In this work, we combined the drug dose prediction model, drug-drug interaction warnings and drugs that potassium raising (K-raising) warnings to create a novel and effective ontology-based assistive prescription recommendation system for patients having both Type-2 Diabetes Mellitus (T2DM) and CKD. Although there are several computational solutions that use ontology-based systems for treatment plans for these type of diseases, none of them combine information analysis and treatment plans prediction for T2DM and CKD. The proposed method is novel: (1) We develop a new drug-drug interaction model and drug dose ontology called DIAKID (for drugs of T2DM and CKD). (2) Using comprehensive Semantic Web Rule Language (SWRL) rules, we automatically extract the correct drug dose, K-raising drugs, and drug-drug interaction warnings based on the Glomerular Filtration Rate (GFR) value of T2DM and CKD patients. The proposed work achieves very competitive results, and this is the first time such a study conducted on both diseases. The proposed system will guide clinicians in preparing prescriptions by giving necessary warnings about drug-drug interactions and doses.

## Introduction

Long-term and sometimes even life-long chronic diseases that directly affect the majority of the world's population are usually asymptomatic, slowly progressing and preventable diseases. Therefore, people suffering from these diseases or groups at risk (age, obesity, etc.) need constant care, monitoring and education [1]. While all the remaining treatments, together with medication, activity and healthy nutrition, are carried out by the patients and their relatives at home, only the patients whose condition worsens are taken under control in the hospitals. Our research specifically focuses on creating a novel and effective ontology-based assistive technology for drug treatments of patients having both Type 2 Diabetes Mellitus (T2DM) and Chronic Kidney Disease (CKD). Because it is vital to treat CKD and T2DM with the right drug and the right dose rate.T2DM and CKD diseases are closely linked to each other.

### Research problem, motivation, and objectives

**Fig. 1:**
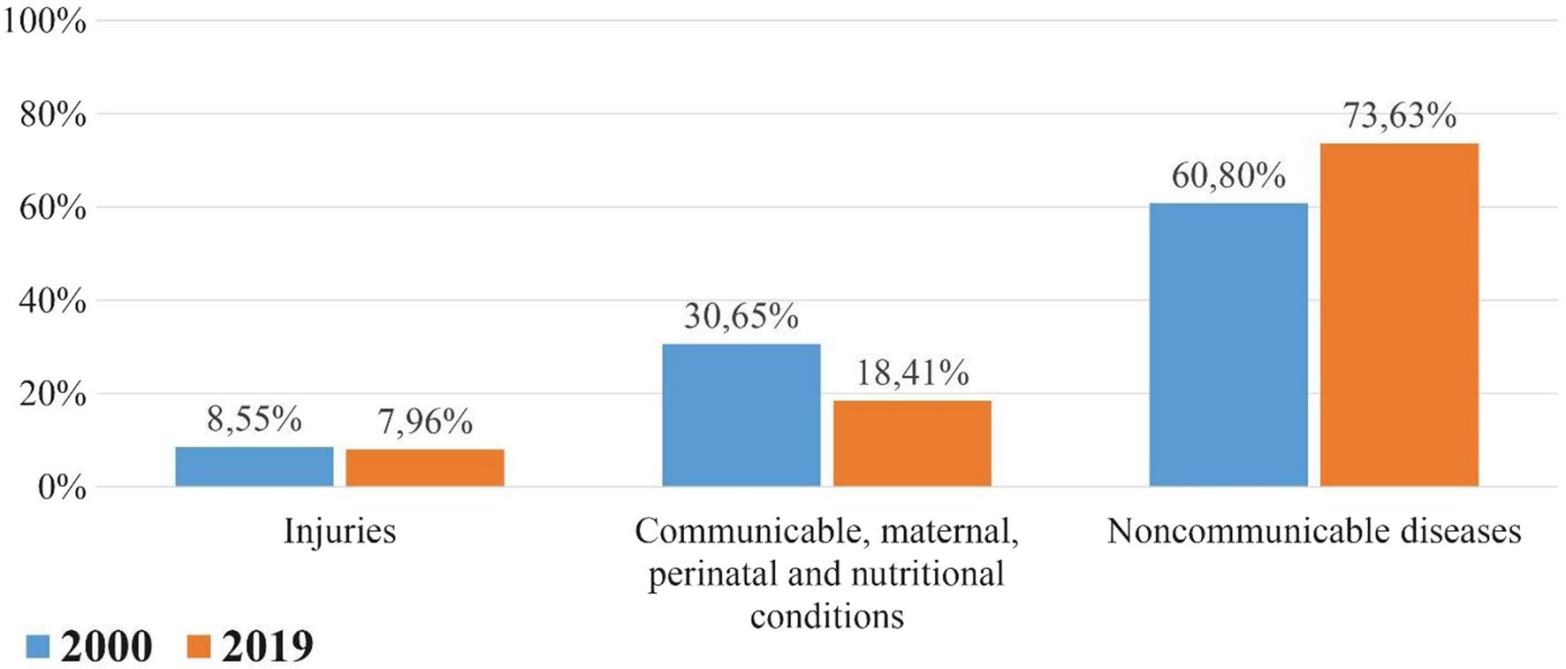
Comparison of all causes of death from 2000 to 2019 with communicable and non-communicable causes of death (authors owns)

According to studies conducted in England and the USA [2], more than 50% of T2DM patients develop CKD. This is because constantly used medications damage the kidneys and ultimately impair their functions. Additionally, 40% of patients with CKD develop T2DM [3]. The reason for this is that the kidneys, whose functions are impaired, cannot filter the waste in the blood properly, and the level of waste urea in the blood rises, preventing the pancreas from producing insulin as required, causing T2DM [4]. Additionally, lifelong medications and treatments used for both diseases (T2DM and CKD) create a great burden on states, and approximately 63% of such patient groups stay in the hospital for much longer periods of time [5].CKD and T2DM, which are interrelated, are our motivation for the subject of our research. An ontology-based, computational assistive technology can be developed to assist specialist physicians in the treatment of patients with both T2DM and CKD. It can be difficult for a specialist physician with a busy work load to memorize the doses of drugs, drug-drug interactions and K-raising drugs used in the treatment of patients with both T2DM and CKD diseases. We try to solve this problem using ontologies and comprehensive semantic rules. With appropriate treatments, patients's quality of life can be improved, hospitalizations can be reduced, and thus mortality rates can be reduced. According to the WHO, when the data for 2019 are compared, it is observed that 7 of the top 10 causes of death are due to non-communicable diseases (NCDs) [4]. When we take the data from the Global Health Data Exchange from 2000 to 2019 and compare all causes of death, it is seen that only the death rates due to NCDs have increased rapidly, while the other causes of death have decreased [6] (Fig. 1). Figure 1 below shows a comparison of death rates for non-communicable diseases, injuries, communicable diseases between 2000 and 2019. Multiple drug prescriptions are issued by different specialist physicians for patients with more than one NCD disease. In patients taking multiple medications, side effects and complications are inevitable as a result of prescribing DDIs, incorrect drug doses, and drugs that K-raising. Therefore, it is vital to consider drug-drug interactions, drug doses, and drugs that K-raising when preparing prescriptions for the treatment of patients with multiple chronic diseases. In this study, the drugs that should be included in the prescriptions of patients with two chronic diseases (T2DM and CKD) at the same time; drug-drug interaction, drug dosage and drugs that K-raising were discussed in detail. According to our research, we found that none of the existing studies have yet proposed a system that provides the necessary scope and integrity by focusing on the interactions of drugs prescribed to patients with both T2DM and CKD simultaneously, drug doses that will vary according to the course of CKD, and drugs that K-raising. Although many ontologies have been developed especially for the treatment and diagnosis of DM and recently for CKD treatments, a new T2DM-CKD drug doses ontology and DDIs ontology is urgently needed for these critical problems, which is the contribution of this paper.

### Proposed approach

Our ultimate goal in this study is to create a new assistive system that can give appropriate warnings in drug treatment based on data obtained from the patient's current status in order to minimize the risks that may occur during T2DM and CKD treatment processes. As a result, people's quality of life can be improved and clinicians can be assisted in the treatment process. This article introduces a novel and effective ontology-based assistive technology for drug therapy of patients having T2DM and CKD. In particular, we propose a new ontology called, DIAKID Ontology. DIAKID was developed using concepts from the Diabetes Mellitus Treatment Ontology (DMTO) and three newly created ontologies. By reusing the DMTO Ontology, we extend it to recommend the correct treatment for all therapeutic knowledge and drug classes. DIAKID calculates the stage of CKD, finds DDI warnings for patients having T2DM and CKD, adjusts drug doses by looking at the patient profile, finds K-raising drugs, and warns physicians and clinicians when necessary.

DIAKID was developed based on research in the literature, specialist physician knowledge and clinical practice guidelines. Specifically, the following new ontologies are developed; Drug-Drug Interaction and K-raising drugs ontology (named as DIAKIDDI), drug doses ontology (named as DIAKIDrugs) and patient profile ontology (named as DIAKIDPatient). To summarize DIAKID ontology is formed from DMTO, DIAKIDDI, DIAKIDrugs and DIAKIDPatient ontologies to provide an ontology-based assistive technology to clinicians. In DIAKID, comprehensive SWRL rules are proposed to alert physicians to patients with both T2DM and CKD; It contains (1) possible T2DM/CKD drug doses that may vary depending on the course of both diseases, (2) DDI interaction warnings, and (3) warnings about K-raising drugs. The main innovative contributions of this work are:

- To present a new ontology-based system that recommends drug treatment plans for patients with both T2DM and CKD, which is the focus and contribution of our research.
- A new ontology, called DIAKID, is proposed that re-uses a modified version of DMTO ontology and introduces new ontologies to create a brand-new treatment system to improve and maintain the quality of life of both T2DM and CKD patients.
- The proposed new ontologies are: A Patient Profile ontology, as well as, DDI, T2DM Drugs, and drug doses ontologies for T2DM and CKD are also proposed that are respectively called DIAKIDPatient ontology, DIAKIDDI ontology, modified DMTO ontology, and DIAKIDrugs ontology.
- Extensive SWRL rules are proposed to warn physicians about patients having both T2DM and CKD; (1) Possible T2DM/CKD drug doses that may vary according to the course of both diseases, (2) DDI interaction warnings and (3) warnings for drugs that increase potassium (K) levels.

To illustrate the effectiveness of the proposed approach, we use a publicly available dataset [7] that contains data about 249 patients having both T2DM and CKD. According to the proposed ontologies and SWRL rules, drug doses, drug interactions, and K-raising drugs are automatically suggested depending on the different stages of T2DM and CKD. The drug doses, drug-drug interactions warnings and k-raising drug warnings that the system recommends specifically for each patient's treatment are independently evaluated by three specialist physicians. Then, the predicted and ground truth data are compared for accuracy, sensitivity and specificity. Results show that the proposed ontology-based treatment approach provides very promising results. Comparing with the previous results in the literature, most of the work provides an ontology-based solution to a single problem such as DM or CKD, whereas the proposed work takes two diseases into account and has a very promising (96–98%) results.

The remainder of the article is organized as follows: Chapter 2 describes the diagnosis and treatment processes of T2DM and CKD diseases. Chapter 3 describes the latest ontology-based developments in DM, CKD, treatments, DDIs, and remote monitoring of patients. Chapter 4 summarizes the outlines and key components of SW technologies. Sect. "Methodology and system architecture" presents the methodology of the proposed system. Chapter 6 details the ontologies developed for the proposed system. Chapter 7 discusses the dataset and its annotation process according to the developed ontologies. Chapter 8 introduces the extensive SWRL rules for DDIs and dose suggestion. Chapter 9 is evaluation and results. Finally, chapter 10 details the conclusions and summarizes future work.

### T2DM and CKD diseases

NCDs are diseases that are not directly transmitted from person to person, and these include Parkinson's disease, diabetes, CKD, Alzheimer's disease, autoimmune diseases, stroke, many heart diseases, many types of cancer, osteoarthritis, osteoporosis, and cataracts [8]. NCDs are the leading cause of death worldwide, caused by factors such as a person's medical history, social life, diet, and environmental factors. NCDs ranked as the highest cause of death among all deaths worldwide; It is responsible for 41 million deaths each year and accounts for 71% of all deaths [9].

There are three different types of DM, which is a non-communicable disease: Type 1 DM, T2DM and Prediabetes [10]. Complex T2DM, which is more directly related to metabolism, is a chronic disease caused by genetic as well as environmental factors [11]. T2DM is a type of diabetes that makes up 90% of all diabetic people in the world and damages organs; It is a condition in which the human body resists and does not respond to insulin, and because insulin cannot work properly, the blood sugar level rises and insufficient insulin is secreted by the body [12]. Diabetes has many complications and some of them are CKD [3] and diabetic retinopathy [13]. The number of patients suffering from diabetic retinopathy and CKD is increasing every year [3, 13].

CKD is a chronic disease that impairs kidney structure and functions. As the disease progresses, wastes reach high levels in the blood, making people sick and causing some complications. These complications can be listed as high blood pressure, anemia (palpitations, dizziness and shortness of breath, etc.), excessive potassium accumulation in the body, malnutrition and nerve damage. In addition to these complications, CKD also leads to the development of heart problems and vascular diseases [14].

### Diagnostic processes

Hemoglobin A1c (HbA1c) is a blood test used as a screening tool to detect type 2 diabetes early, and the test result represents 2- or 3-month average blood glucose concentrations [15]. T2DM can be diagnosed by looking at plasma glucose or HbA1c values. Table 1 gives the result of the diagnosis of type 2 diabetes mellitus according to the value given by the HbA1c test result.

**Table 1:** T2DM diagnosis based on HbA1c value [83]

Hemoglobin A1c	T2DM Diagnosis
HbA1c < 5.7%	Normal range
5.7% < HbA1c < 6.4%	Prediabetes
HbA1c > 6.4%	Diabetes

A person's GFR is calculated by doctors using age, sex, and test results to determine kidney function and stage of disease. To detect asymptomatic kidney disease, specialist physicians estimate GFR (eGFR) using a formula [16]. According to the results of the GFR test, the stage of chronic kidney disease and the functioning level of the kidney are shown in Table 2. As the GFR value decreases, kidney functions deteriorate proportionally.

**Table 2:** CKD stages and explanations by eGFR level [16]

Stage	Description	eGFR (ML/MIN/1.73 M^2^)	Kidney function
S1	Possibility of kidney damage and normal kidney function	eGFR ≥ 90	90–100%
S2	Kidney damage and initial loss of kidney function	60 < eGFR < 89	60–89%
S3a	Mild and moderate loss of kidney function	45 < eGFR < 59	45–59%
S3b	Moderate and severe loss of kidney function	30 < eGFR < 44	30–44%
S4	Severe loss of kidney function	15 < eGFR < 29	15–29%
S5	Kidney failure	eGFR < 15	Less than 15%

### Treatment processes

In order for the treatment of T2DM to be successful and give good results, it is necessary to make changes in nutrition and lifestyle (diet and sports). In order to significantly reduce the risk of many diseases (ischemic heart disease, hypertension, neuropathy, retinopathy, nephropathy, etc.), glycosylated hemoglobin should be close to normal. There are two types of drug treatment for T2DM, oral and injectable. Metformin is offered by physicians as a first-line treatment option for many patients [17]. Although treatments for CKD are limited, appropriate treatments can slow their patients' progression to end-stage kidney disease. Blood pressure control of patients is achieved in the treatment of CKD by using ACE-i (Angiotensin Converting Enzyme inhibitors) or ARBs (Angiotensin II Receptor Blockers) [18].

### Drug dose and DDIs

Physicians may prescribe more than one drug in order to slow down the course of DM and improve the quality of life in a diabetic patient. This can increase the likelihood of DDIs, put the patient's life at risk, or impair quality of life. Doctors should be mindful of DDIs when prescribing anti-diabetic agents. Most drugs are related to metabolism, but caution should be exercised when prescribing drugs that impair renal status as they are excreted via the kidney [19]. Physicians should prescribe drugs by considering age, body mass index, genetic status, blood, urinalysis results, and other diseases, if any when adjusting drug doses for T2DM patients [20].

Patients with CKD often have more than one drug prescription. Interactions of drugs prescribed for this patient group are a common problem and can lead to serious side effects and complications if not detected early. Therefore, physicians should be careful when prescribing drugs with potential interactions to prevent adverse events that may occur as a result of DDIs [21]. Adjusting the drug dose in patients with CKD is as important as DDI and can cause adverse effects and adverse outcomes. Physicians should specify doses based on the patient's eGFR when prescribing drugs [22].

### Related work

Recently, SW-based smart solutions have become popular for the diagnosis, monitoring and treatment of T2DM, CKD, as well as DDI analysis for patients with both T2DM and CKD. We briefly discuss related Semantic Web-based solutions for T2DM and CKD (in Sect. "Ontology-based systems for T2DM and CKD") and T2DM-CKD DDI approaches (in Sect. "T2DM and CKD Drug-drug interaction approaches"). Finally, in Table 3 the reviewed related works are also summarized in detail comparing to their ‘Aim’, ‘Developed Ontologies’ and ‘Reused Ontologies’.

**Table 3:** Compare of created and reused ontologies

Source	Aim	Developed Ontologies	Reused Ontologies
El-Sappagh, S. H. et al. 2014 [43]	Diabetes mellitus diagnosis	Case base ontology	BFO: Basic Formal Ontology
Brochhausen, M. et al. 2014 [57]	Tracing the evidence underlying DDI knowledge	DIDEO	ChEBI: Chemical Entities of Biological Interest
El-Sappagh, S. et al. 2014 [31]	Diabetes mellitus diagnosis	Domain Ontology	CMO: Clinical Measurement Ontology
Herrero-Zazo, M. et al. 2015 [58]	Prediction of DDIs (DDIs)	DINTO	DDO: Diabetes Diagnosis Ontology
Hempo, B., et al. 2015 [42]	Providing personalized care for patients	DKOs	DINTO: The Drug-Drug Interactions Ontology
El-Sappagh, S., & Elmogy, M. 2016 [33]	Diabetes diagnosis	Fuzzy ontology-based CBR framework	DMTO: Diabetes Mellitus Treatment Ontology
El-Sappagh, S., & Ali, F. 2016 [35]	Facilitating problem-solving in diabetes management	DDO	DOID: Disease Ontology
Mahmoud, N., & Elbeh, H. 2016 [53]	Individualizing the treatment of T2DM	Patient profiles ontology Anti-diabetic drug ontology	DrOn: Drug Ontology
Sherimon, P. C., & Krishnan, R. 2016 [41]	Evaluation of risk factors and appropriate treatment recommendation in patients with diabetic nephropathy	Diabetic Patient Clinical Analysis Ontology Semantic Profile Ontology	FOAF: Personal Profile Ontology
Zhang, Y. F., et al., 2017 [23]	Personalized patient follow-up evaluation	Ontology-based	FoodOn: Food Ontology
El-Sappagh, S., & Elmogy, M. 2017 [36]	Diabetes diagnosis	CBRDiabOnto	GO: Gene Ontology
Chen, R. C. et al. 2017 [54]	Helping clinicians manage T2DM when choosing antidiabetic drugs	Drug knowledge ontology	I2B2: Informatics for Integrating Biology & the Bedside Ontology
Martinez-Millana, A., et al. 2018 [34]	To provide a technological structure where research and decisions can be made for patients with T2DM	Common Ontology	IAO: Information Artifact Ontology
Kang, Y. et al. 2018 [56]	Identifying the special needs of CKD	DSOAE	ICNP medicine ontology
Guermah, H. et al. 2018 [44]	Dealing with CKD problems	SVM Context Ontology	KTAO: Kidney Tissue Atlas Ontology
Abd Elkader, S. et al. 2018 [46]	Diagnosis of Chronic Kidney Disease	Kidney Disease Ontology	MBCO: Molecular Biology of the Cell Ontology
Alian, S. et al. 2018 [24]	Creating a personalized diabetes recommendation system	Ontology-based Knowledgebase	OAE: Ontology of Adverse Events
Okikiola, F. M. et al. 2018 [38]	Assisting in diagnosing diabetes, prescribing treatment and medication	Ontology-based Diabetes Management System	OBI: Ontology for Biomedical Investigations
El-Sappagh, S. et al. 2018 [37]	Diabetes Diagnosis	Diabetes complications and symptoms ontology	OBO: Open Biomedical Ontology Foundry
Cole, N. I. et al. 2018 [47]	Diagnosis of Chronic Kidney Disease	CKDO	OGMD: ontology for glucose metabolism disorder
El-Sappagh, S. et al. 2018 [40]	Customized T2DM Treatment	DMTO	OPMI: Ontology of Precision Medicine Investigation
Ali, F. et al. 2018 [30]	Suggesting specific prescriptions	Fuzzy ontology	PATO: Phenotypic Quality Ontology
Nachabe, L. et al. 2018 [27]	Helping patients with diabetes in their daily life	Smart Diabetes REFerence (SDREF) Ontology	RO: Relation ontology
Xu, E. et al. 2019 [55]	Compliance with antidiabetic drug therapy in T2DM patients	Drug Ontology	RxNorm Ontology
Song, X. et al. 2019 [45]	Improving Diabetic Kidney Disease risk estimates	Ontology-based	SMASH: Semantic Mining of Activity, Social, and Health data
Chen, L. et al. 2019 [39]	Deciding on the diagnosis of diabetes	OMDP	SNOMED CT: Systematized Nomenclature of medicine-clinical terms
Mallika, C. et al. 2019 [52]	Early diagnosis of diabetes and providing up-to-date information	Ont-SVM	SSN/SOAS ontology sensor network ontology
Titi, S. et al. 2020 [28]	To monitor patients	Fuzzy Ontology	Symptom Ontology
Bravo, M. et al. 2020 [32]	Diabetic patient profiles management	Diabetic Patient Profiles Ont.	Time Ontology
Madhusanka, S. et al. 2020 [25]	Knowledge representation of clinical guidelines on diabetes	Ontological Clinical Decision Support System	Virtual Medical Record (vMR) Ontology
Ong, E. et al. 2020 [48]	Providing a comprehensive map of the kidney	KTAO OPMI	
Elhadj, H. et al. 2021 [29]	Advising on the care of patients and the preparation of their prescriptions	Do-Care ontology	
Nisheva-Pavlova, M. et al. 2021 [26]	Improving the diabetic patient's lifestyle	–	
DIAKID ontology	Advising clinicians by giving warnings for DDIs and drug doses when prescribing for multi-prescriptions	DIAKID ontology (Patient ontology, Drug ontology and DDI ontology)	

### Ontology-based systems for T2DM and CKD

Recently, SW-based smart solutions for kidney and diabetes disease research have become popular. All studies reviewed in this area recommend at least one ontology for kidney and diabetes research or reuse existing ontology(s). These ontologies differ in purpose: diagnosis, treatment, research, knowledge base building, disease management, etc. As a result, ontologies have been developed or reused to improve patients' quality of life, treatment modalities, diets, activities, and early diagnosis. Related works in these fields are as follows:

Ontology-based and computer-interpreted CDSS (Clinical Decision Support Systems) have been developed and used for personalized chronic disease management that requires continuity: disease management using real data from T2DM Patients [23], a healthy lifestyle recommendation for American Indians (AI) diabetes patients [24], formulating a number of published clinical decisions for diabetic patients in Sri Lanka [25], and making dietary recommendations to diabetics [26] by producing more precise recommendations.

Recently IoT (Internet of Things) based semantic solutions are proposed. [27] uses a semantic system consisting of seven ontologies and IoT devices; IoT devices provide information about patients by monitoring parameters such as daily activities, meals and symptoms using wearable, burnable or usable devices. A fuzzy ontology-based diabetic monitoring system is also proposed by. [28] Proposes a system by applying a set of SWRL rules to determine the patient's current health status such as Normal, Abnormal or False. Captured by IoT inputs, and a type 2 fuzzy ontology-based system [29], researchers efficiently monitor the health status of diabetes patients who are under risk using IoT-based healthcare services. To provide appropriate prescription and diet lists, [30] proposes a remote healthcare monitoring system to assist diabetics in their daily lives.

Many other ontology-based systems have been developed or reused in order to facilitate the lives of diabetes patients and to assist clinicians in the diagnosis and prediction of diabetes. These ontology-based systems can be summarized as follows. [31] Diagnosis of diabetes mellitus based on the conceptual model of SNOMED CT (Systematized Nomenclature of medicine-clinical terms), [32] allows for managing the patient profile and deciding on a clinical diagnosis of diabetes. [33] Provides a fuzzy-based CBR (Case-Based Reasoning) framework for the diagnosis of diabetes on case-by-case basis. [34] The new system, which uses different artificial intelligence tools by integrating them into the daily routine, and the system established in the clinical environment and using clinical databases, evaluated 7 patients with different Type 2 Diabetes models. [35] Presents the Diabetes Mellitus Diagnostic Ontology (DDO) system, which represents an important work in the development of next-generation patient-centred decision support. [36] Also introduces a system called CBRDiabOnto that aims to advance CBR research for diabetes diagnosis by proposing a fuzzy-based ontology. [37] Proposes a Fuzzy Rule-Based Systems (FRBS) framework based on the standard ontology of SNOMED CT, which uses many aspects of ontology reasoning and the FAHP (Fuzzy Analytical Hierarchy Process) for diabetes diagnosis, and is a new semantically interpretable framework. [38] develops a Bayesian Optimization Technique and ontology-based diabetes mellitus knowledge base for drug suggestion, diagnosis and prescribing.

Recent studies for the follow-up and control of the treatment processes of diabetes patients aim to increase their quality of life and help clinicians by suggesting appropriate treatment opportunities for patients. [39] Proposes an Ontology-Based Model (OMDP) model that reuses 6 ontologies to gather detailed information about diabetes for diagnosis and useful treatment. [40] Develops an extensive DMTO, which provides proof of concept by reusing 11 ontologies for modelling TPs of patients with T2DM. Another decision support system is proposed by [41] called OntoDiabetic which consists of 7 ontologies. The aim of the study is to predict the risks that diabetes patients may experience and to recommend appropriate treatments. [42] Diabetes knowledge-based ontologies (DCOs) were developed by creating patient profiles to offer personalized self-care for diabetes patients, [43] for diet and medication plans for diabetes treatment using CPGs (Clinical Practice Guidelines) and EHR (Electronic Health Record) content It aims to provide basic concepts. Ontologies also aim to [44] integrate patient data from personal health devices and wearables and provide up-to-date information to physicists by enabling early detection of diabetes.

Compared to the active field of research on diabetes, fewer ontologies and systems have been proposed in the CKD field to provide patients with a quality life and also to assist clinicians in the early diagnosis and treatment of CKD. [45] Introduces a system focusing on Linear Support Vector Machines to perform classification and prediction of CKD problems that are particularly difficult to diagnose. [46] Utilizes an unbiased data-based review using Electronic Medical Records (EMRs) to identify potential Diabetic Kidney Disease (DKD) patients 6 months prior to baseline. [47] Recommends a methodology for CKD diagnosis based on ontological reasoning mechanism using a series of data mining algorithms. [48] developed a field ontology created using primary health data routinely collected from the RCGP&RSC (Royal College of Practitioners Research and Surveillance Center) to find CKD cases. The field ontology is developed using reference ontologies and the KPMP (Kidney Precision Medicine Project). Using this ontology, specifically patients receiving kidney transplant and dialysis therapy or CKD stage 1 or 2 are aimed to be identified. Finally, [49] develops a system to improve the analysis of kidney-related data, support precision medicine and find solutions to CKD problems.

Although there are related works individually focusing on either diabetes or CKD using ontologies, none of the works focus on TPs for patients having both T2DM and CKD. 90% of people with diabetes have T2DM [50]. Studies show that more than 33.2% of people with T2DM also have CKD [51]. Therefore, there appears to be a significant gap when the literature is searched to examine and analyse TPs for patients with both T2DM and CKD, which is the novelty and focus of the proposed study. Another important issue is that the drugs used in T2DM can worsen CKD or have the opposite effect [52]. This is called the T2DM and CKD DDI problem. In our proposed system, we also focus on solving this problem and offer an ontology-based solution for precise drug dosage prescriptions according to the stage of patients with both T2DM and CKD. The next section discusses related works for the DDI problem.

### T2DM and CKD Drug-drug interaction approaches

Nowadays ontologies have begun to be used in DDIs, side effects of DDIs on patients, negative consequences of DDIs and so forth. In these studies, ontologies are developed or reused to improve the quality of life of people struggling with chronic diseases. We observe that many studies on DDIs mostly focus on diabetes patients. The aim of these works is to prevent the negative effects of DDIs on the patient and to help clinicians in the preparation of prescriptions. For example, [53] establishes patient profiles ontology and anti-diabetic drug ontology (IRS-T2D). Using the system, a drug dosage guide is created to individualize HbA1c target, drug selection, and T2DM treatment. Based on the ADA (American Diabetes Association) and the EASD (European Association for Diabetic Research) for HbA1c target and antidiabetic drug recommendation, TOPSIS (Technique for Order Preference by Similarity to Ideal Solution) [54] calculates the rank of antidiabetic drugs to help clinicians manage T2DM when selecting antidiabetic drugs. [55] proposes a framework for drug compliance assessment and data analytics by creating a drug ontology and T2DM patient cohort using EHR data. The aim is to provide data for diabetes prevention and treatment. [56] develops a knowledge base for CKD using specific Disease-Specific Adverse Events Ontology (DSOAE). The knowledge base is created and implemented using data from different sources using their proposed DSOAE to study the specific needs of CKD.

Other two developed systems are for DDI are; 1) [57] DDI Evidence Ontology (DIDEO), proposed to avoid conflicts between drug information sources used by clinicians, conducts studies for the first step towards developing a new domain representation to allow monitoring of evidence underlying potential DDI (PDDI) information and explains. 2) [58] Ontology for DDI Knowledge (DINTO) estimates DDI by incorporating all the information from different DDIs, including PK (pharmacokinetics) and PD (pharmacodynamics), rather than focusing on the DDI mechanism for a particular field or disease.

**Fig. 2:**
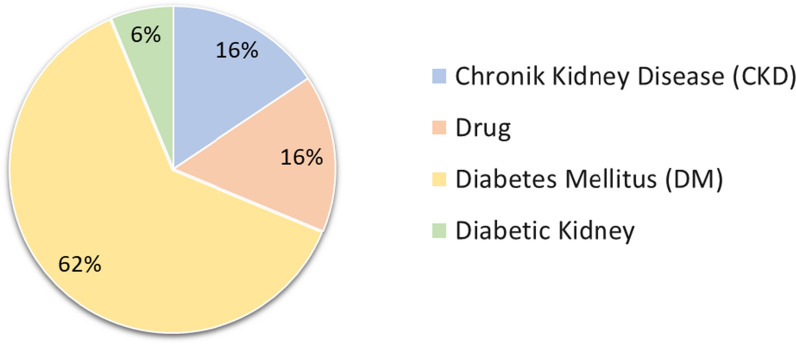
Comparison of ontologies according to their purpose (authors owns)

**Fig. 3:**
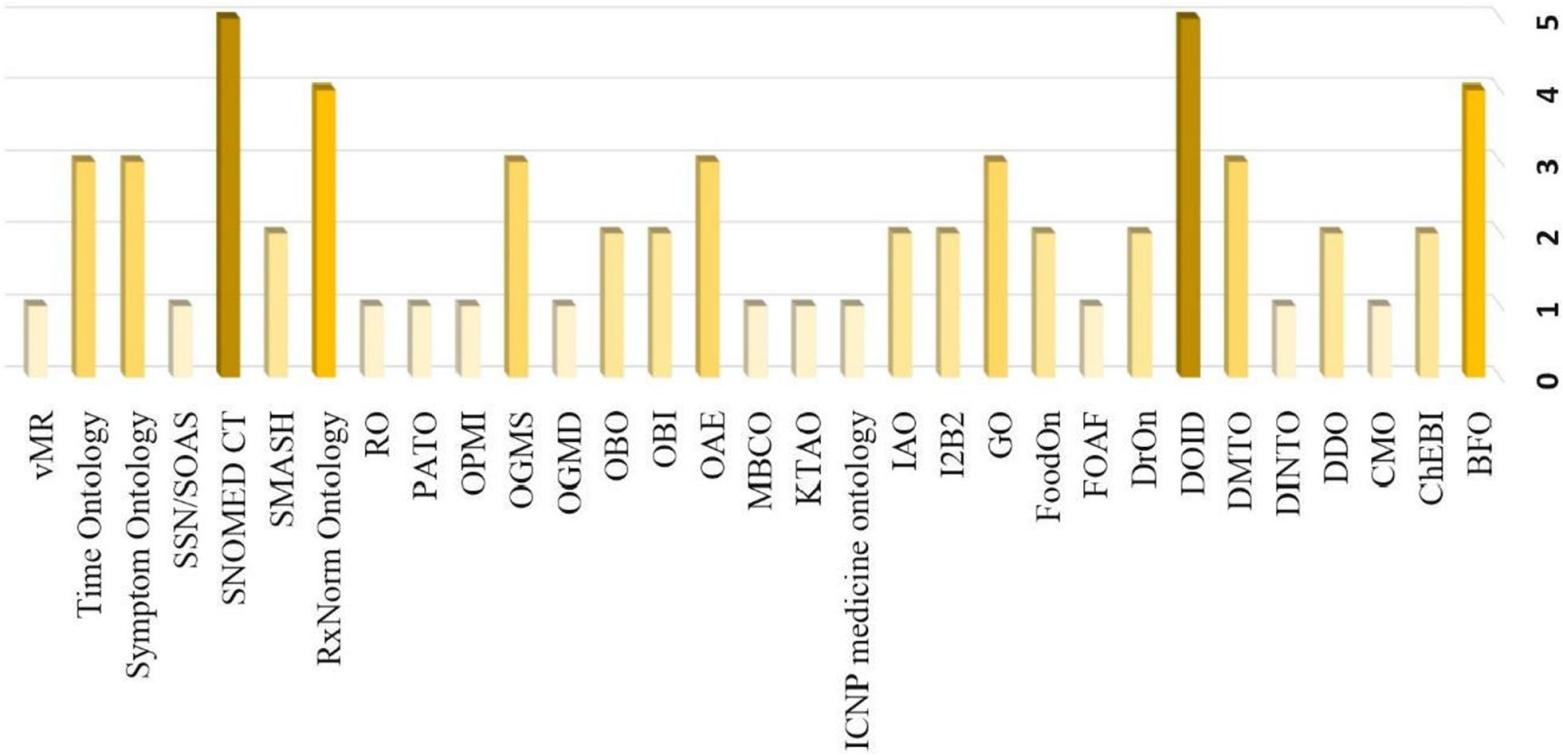
Reused ontologies—among the reviewed 33 number of papers (authors owns)

In Table 3, we summarize related works for diabetes, CKD and DDI. It is observed among these three fields, research on diabetes is more popular. However, there is a gap in the literature; There is no system to provide TPs or prescribing information regarding DDIs and doses for patients with both T2DM and CKD. In this study, we focused especially on this problem, which is the novelty of our approach. When all studies summarized in Table 3 presented as graph as shown in Fig. 2, we observed that DM studies were more popular than studies on Drug-Drug Interaction and CKD. In our literature review, we observed that many ontologies are reused. When we graphed these results as in Fig. 3, we saw that DOID and SNOMED BT were used in most of the studies.

### Semantic web technologies

The primary purpose of SW is to make data machine-readable, and to achieve this vision it provides a common framework that allows content, metadata and other information to be shared and reused across applications, organizations and communities [59]. An extension of the World Wide Web, SW is a collaborative structure of a collection of technologies that give information well-defined meanings to enable computers and humans to work collaboratively more efficiently and effectively.

SW technologies make it possible for us to identify and understand relationships (links) between data on the web, unlike traditional web pages. In traditional web pages, hyperlinks describe the relationship between the current page and the target page, whereas in SW such relationships can be established on the Web between any two concepts that have unique identifiers (i.e. resources) [60]. Also, relationship types can be expressed in detail in SW.

**Fig. 4:**
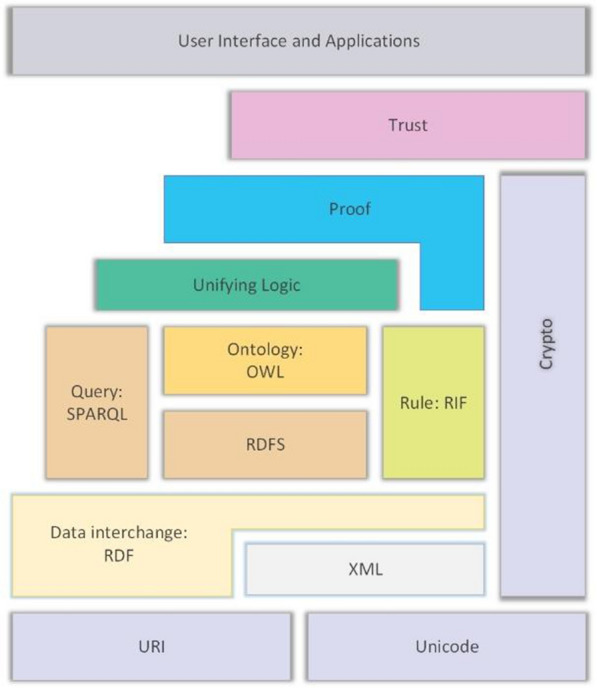
SW-Technologies Stack

As seen in Fig. 4, technologies start at the lowest layer on the Internet, with URI and Unicode. Above this layer is the information exchange layer, which includes XML and RDF. On top of this is the Resource Description Framework (RDF). Above this layer is OWL, which is the Web Ontology language, or RDFS, which is the RDF schema language. One of the core components of SW is RDF, and RDF allows generating machine-processable metadata. Another building block of SW is the URI (Uniform Resource Identifier). Every accessible, viable, and maintainable content in a SW context has a unique URI. If different data use the same URI, it means they are using the same resource [61].

**Fig. 5:**
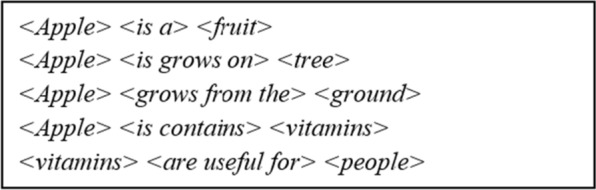
Sample RDF Triples (informal example)

The RDF expression structure is as follows [62]: < *subject* >  < *predicate* >  < *object* > . Some triple samples are given in Fig. 5 below.

**Fig. 6:**
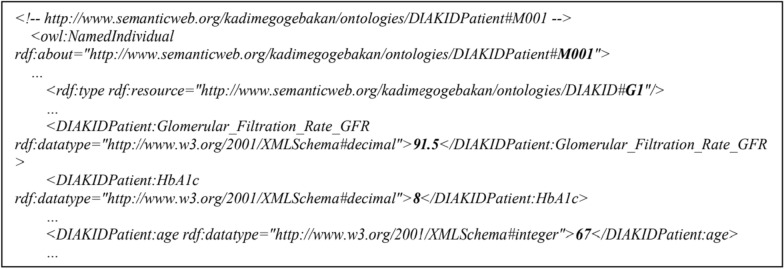
RDF/XML Sample Metadata

RDF data can also be serialized and stored in various formats such as JSON-LD (.json), N-Triples (.nt), Turtle (.ttl), and RDF/XML (.rdf). RDF/XML sample metadata is shown in Fig. 6. Resource Description Framework Schema (RDFS) provides a set of classes that provide a data modelling dictionary for RDF data, providing an extension of the simple RDF dictionary on the Web and a general-purpose language for building ontologies [63]. RDFS, used to group resources, is used as the rdfs:class predicate and is defined by a directed graph labelled node and edge [64].

The knowledge representation language OWL (Web Ontology Language) used to write and develop ontologies is an extension of RDF and RDFS. OWL, the modelling language in SW applications, converts everything in descriptive logic, while RDF and RDFS do not. It defines the OWL, OWL Lite, OWL DL and OWL Full layers, which define the concept of layer providing better features for rollback purposes [65].

Ontology provided by knowledge collections is the third fundamental building block of the Semantic Web. Acting as a document or file for AI and Web researchers, an ontology formally defines relationships between terms. An ontology in RDF format using OWL provides more detailed descriptions of classes, properties, class hierarchy, individuals, and constraints in a given domain [66].

**Fig. 7:**
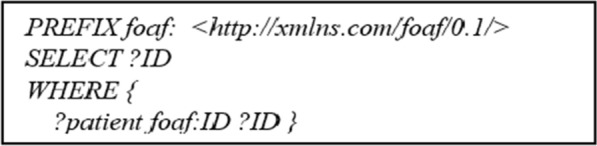
A Sample SPARQL Query

SPARQL is a query and protocol [67] of SW for search and retrieval. SPARQL uses SQL-like statements to query RDF data. An example SPARQL query is given in Fig. 7 below.

The proposed SWRL for SW is used to infer implicit knowledge and is syntax based on a combination of OWL Web Ontology Language's Rules Markup Language, OWL DL, and OWL Lite sublanguages [68]. The SWRL syntax extends the abstract syntax to provide its formal meaning as described in the OWL Semantics and Abstract Syntax document.

SW can be applied in many application areas, from Web user interfaces to surveillance, e-learning and healthcare systems. In healthcare, software technologies are used to enable interoperability [69], diagnosis and treatment, procedural creation, cost reduction, integration, and data reuse.

## Methodology and system architecture

### Methodology

**Fig. 8:**
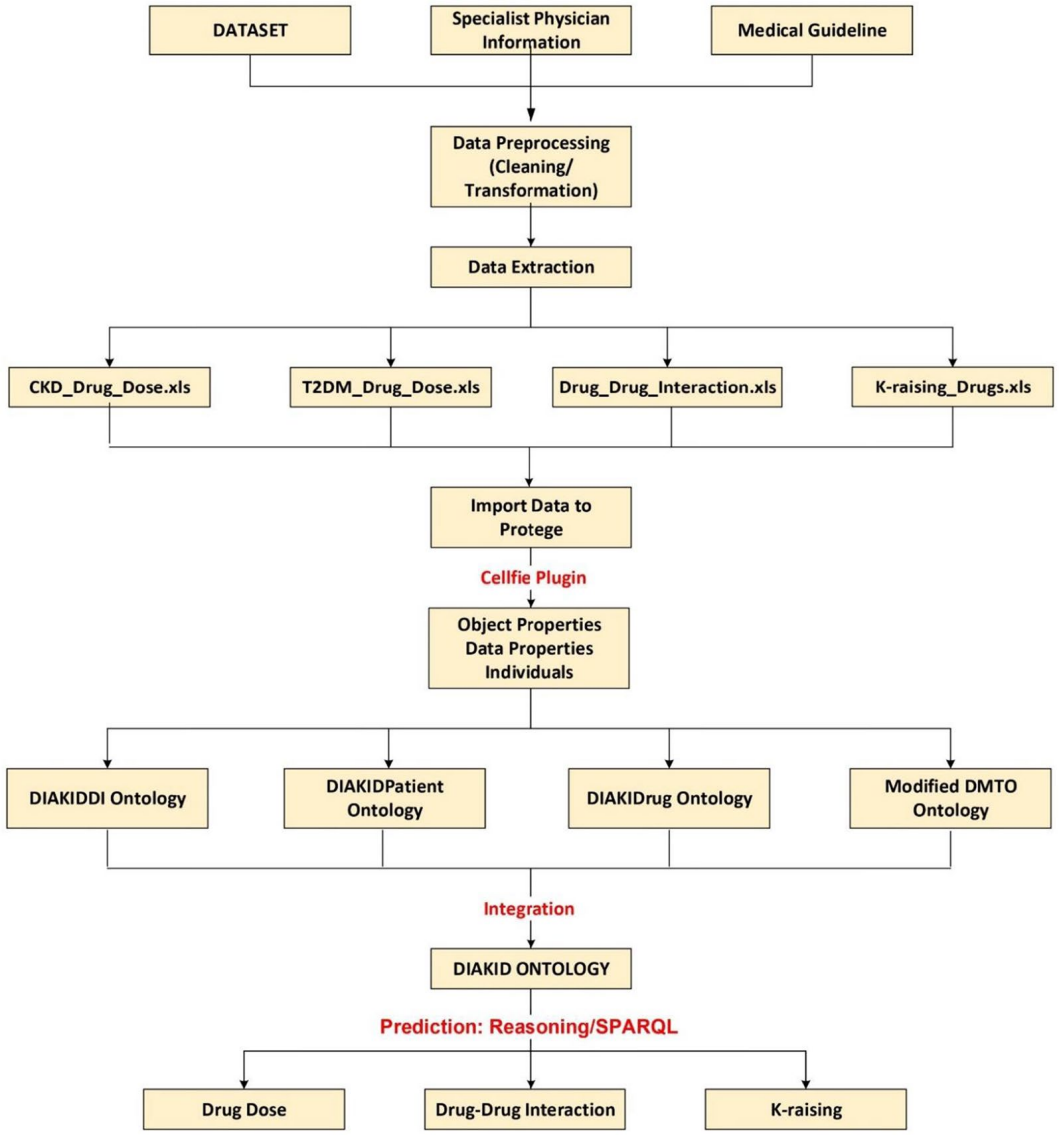
Methodology illustrating the dataset gathering stages and integration of data into the DIAKID ontology

In the proposed DIAKID system, all drug classes, drug doses and patient characteristics are linked to the patient identity and all data is customized for each patient. While creating the DIAKID system, the following procedure was used:

- **Finding a dataset, an expert knwoledge and clinical guidelines:** A publicly available data set was found to test the proposed system on patients. Then, an expert physician was found to guide the creation of drug lists as well as relations between drugs used in the treatment of T2DM and CKD. Finally, we followed the clinical guidelines [70] while creating the DIAKID ontology and comphrehensive semantic rules.
- **Organizing the dataset and populating DIAKID ontology:** The dataset contains 41 characteristics (age, BMI, hbA1c, etc.) of 249 patients in Excel format [7]. However, the data on the active pharmaceutical ingredients of all drugs used in the treatment of T2DM and CKD were created by the authors was in semantic format (OWL) according to the information obtained from specialist physician and medical guidelines. Therefore, the dataset was organized into lists (patients, drugs, drug-drug interactions and K-raising drugs) in order to populate data in the proposed DIAKID ontology and data sets were obtained and transferred to Protégé. This was achieved by writing scripts with the help of Celfie, where the data in Excel was transferred to the object property, data property and individuals of the ontologies. As a result of the transfer, four ontologies were obtained: DIAKIDDI, DIAKIDPatient, DIAKIDRug and Modified DMTO. All ontologies then were combined under the umbrella of the DIAKID Ontology.
- By creating comprehensive SWRL rules; A unique system was created that can recommend drug doses and provide DDI and K-raising drug warnings. In the created system, all drug classes, drug doses and patient characteristics are linked to the patient identity and all data is customized for each patient. Figure 8 is the summary of the DIAKID system that has been created.

The unique DIAKID system will guide specialist physicians and clinicians when writing prescriptions to patients. When specialists prescribe drugs to patients, the system will give necessary warnings about drug dosage, DDI and K-raising drugs. Ultimately, our aim is to improve the quality of life of patients and reduce the burden of specialist physicians with a busy work schedule by preventing errors caused by incorrect drug dosage, DDI and K-raising drugs.

### Structure of the recommendation system

**Fig. 9:**
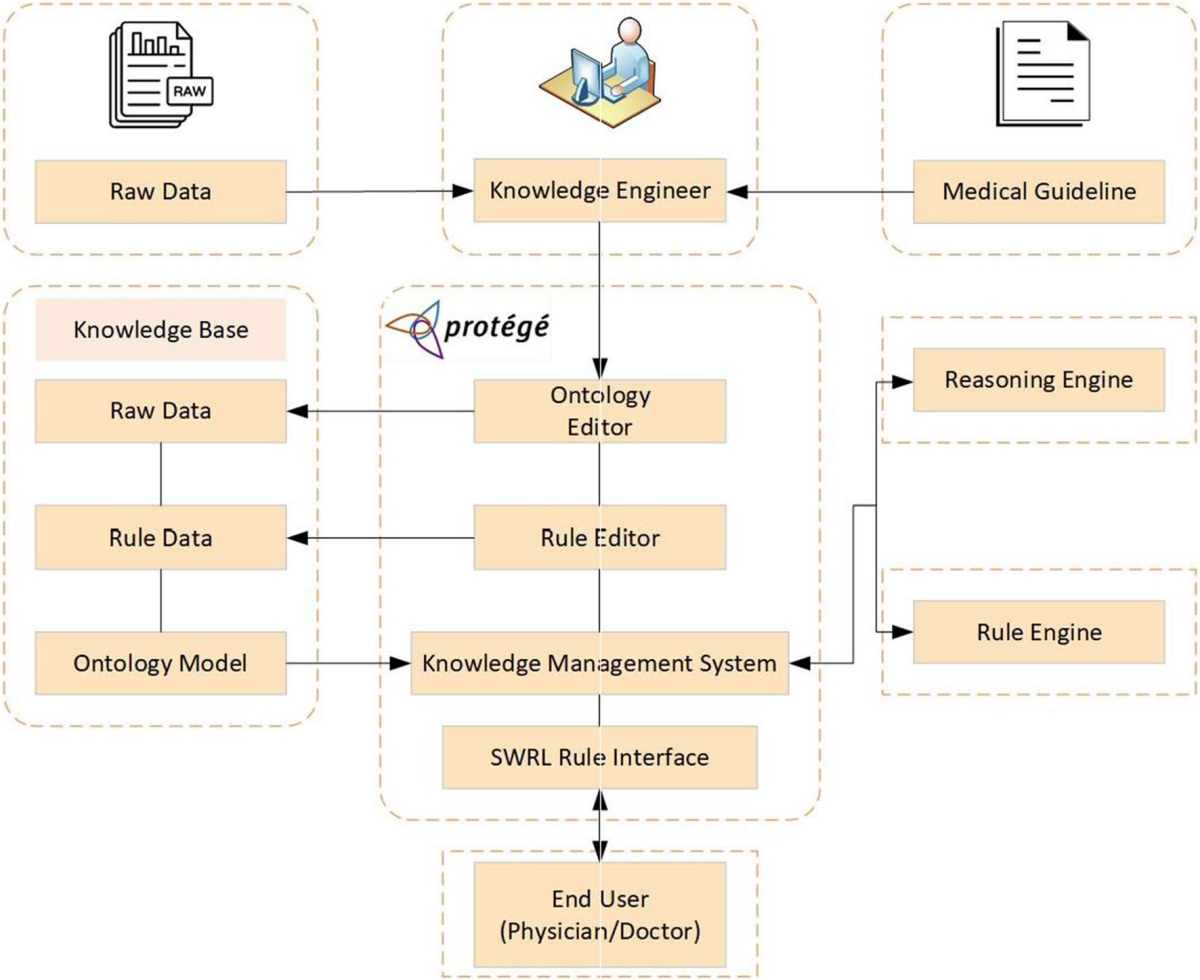
The structure of recommendation system for drugs

The KDIGO (Kidney Disease Improving Global Outcomes) 2020 Clinical Practice Guideline [70] and specialist physician information were used as criteria for the development and management of clinical practice for T2DM and CKD. CKD drug ontology and DDI ontology were systematically created with the help of a nephrologist. The drug ontology used in the treatment of T2DM was obtained by reusing the DMTO ontology and this ontology did not need to be restructured. We've expanded existing drug classes and added individuals. In addition, with the advice of a specialist doctor, all drug classes and doses were created in Protégé according to CKD stages. DIAKID includes four modules: dataset (raw data), ontology management system, rule editor, and query editor, as shown in Fig. 9. The dataset is the primary core part of DIAKID where core data can be saved as Ontology Web. The ontology management system allows editors to create and update the ontology model. Protégé 5.5, an open-source ontology software, was used to implement the development of DIAKID. The rule editor is often used to edit and run the Semantic Web Rule Language rules of the ontology model.

**Fig. 10:**
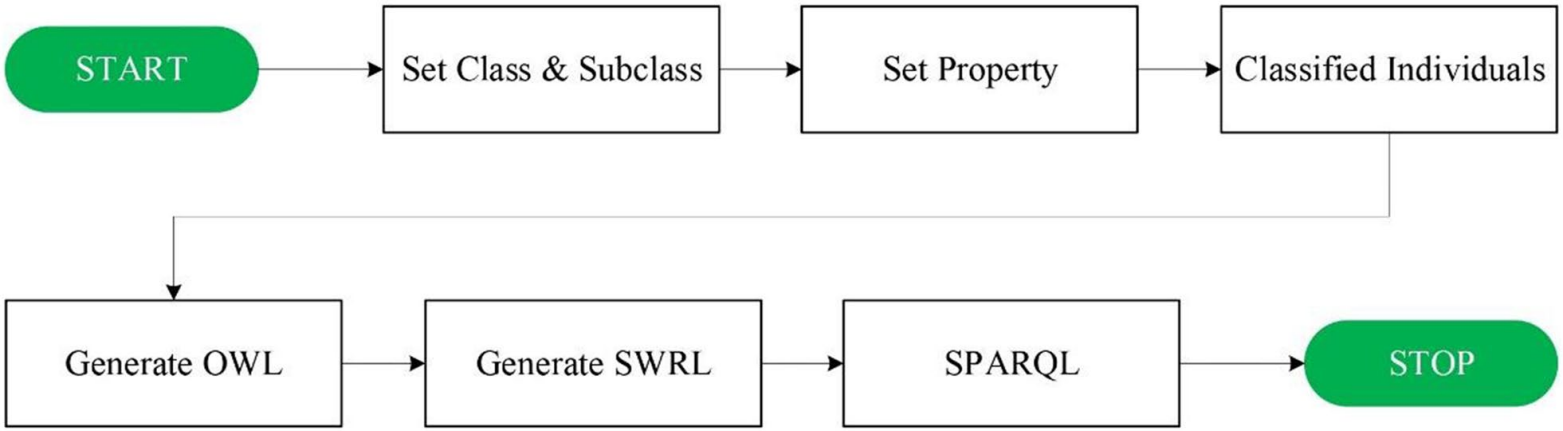
Flowchart of recommended system

DMTO and newly created drug-drug interaction, drug dosage determination, and patient ontologies were used to support the rule framework of the DIAKID ontology. Protégé was utilized to manage created ontologies, knowledge base and T2DM-CKD drug suggestions. An ontology is essentially built on three basic elements: classes, attributes, and relationships. Classes are used to represent domain information. Attributes are used to define classes, and relationships are used to describe the connection between classes. The flowchart of our system is as shown in Fig. 10: First, the classes and subclasses are created. Then, properties are created, followed by the creation of individuals based on the created ontologies. To infer the drug warnings, SWRL rules are created and executed. Finally, with SPARQL, inferred axioms are visualized.

### Challenges

When we started this research, we encountered several difficulties during knowledge and data gathering. Two of the challenging issues were: (1) Finding physicians who specialized in both T2DM and CKD diseases. After contacting to several specialists in the field, we managed to find specialists and use their expertise to generate extensive SWRL rules for drug dose prediction, DDI warnings and K-raising drugs. In addition, for evaluating the predictions of the proposed system, we asked these specialist doctors to assess the accuracy of the system predictions. (2) The second challenge was finding an open dataset that contains patient data about T2DM and CKD-related test results. After several searches, we managed to find the open dataset [7] and re-purposed the dataset to this work as we explained above. To summarize, the proposed work requires expert knowledge and patient data for its predictions and it can be challenging to obtain them.

### DIAKID: Proposed ontology

Ontologies, also known as the knowledge base, which form the basis of many smart applications and systems today; It is a collection of statements written in a language such as RDF and OWL that describe relationships between abstract ideas and set logical rules (SWRL) for reasoning about concepts. Linking or reusing ontologies has enormous benefits in medicine as it supports semantic interoperability between different datasets and applications, improves accuracy, and reduces engineering costs and efforts. At DMTO Ontology, we've expanded the list by adding classes and subclasses to drugs. We are removing it from ontology as the drug Troglitazone in DMTO has been withdrawn from the market [71].

Our ultimate goal in this study is to create a unique system using SW technologies that can provide appropriate warnings during the prescription of drugs for T2DM and CKD patients based on available patient data. This can minimize the risks of DDIs and inappropriate drug dosages that may occur in the treatment processes for T2DM and CKD. As a result, our aim is to increase the quality of life by suggesting a useful system for the patient's health and to suggest a system that can help clinicians in the treatment process. We develop patient ontology and drug ontology for the system that we propose and combine TPs and DDIs in the DIAKID ontology. And we also create the DIAKID Ontology by reusing the DMTO. Therefore, the proposed DIAKID ontology is new and aims to assist patients and physicians by solving the problems of K-raising drugs, DDIs, and drug doses for T2DM and CKD patients.

### Reused DMTO ontologies

Class, Object Properties and Data Properties of DMTO: We reused the previously created ontology to develop our own ontology. The DMTO ontology was reused for diabetes diagnosis, treatment, and diabetic DDI. This ontology was more useful and better served our ultimate goal, the treatment and care of diabetic patients with CKD. In our work, we particularly preferred DMTO ontology since it contains diabetes drugs and their active ingredients. The classes, data properties and object properties, created from DMTO for the interactions of drugs used to create TPs of T2DM patients and drugs used in treatment, were integrated into DIAKID.

The main goal of our research is to reveal the interactions of drugs with each other in patients with more than one prescription and to help physicians/doctors in adjusting the drug dose according to the condition of the disease. Our proposed ontology-based system makes it easy for field experts to learn about DDI and also helps to warnings experts to protect patients from the side effects of DDIs and improve people's quality of life. We developed DIAKID by applying the newly created ontologies and reused ontologies together, which we will explain one by one below.

### Patient ontology

**Fig. 11:**
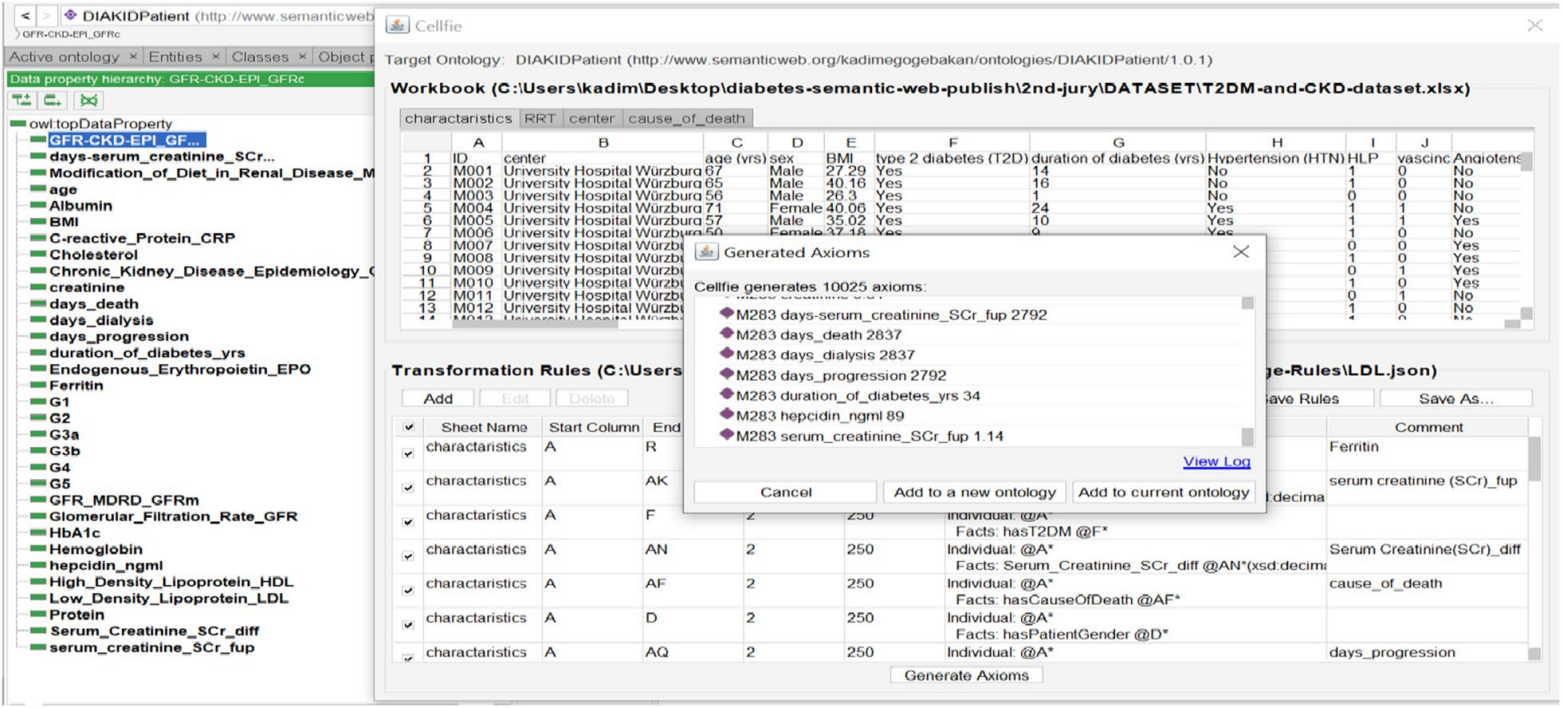
Patient information transferred and stored in Protégé with the help of Cellfie

The patient ontology consists of 41 characteristic features of 249 patients transferred from Excel format to Protégé with the help of Cellfie and stored [72]. Cellfie is a Protégé Desktop add-on for importing spreadsheet data from Excel format into OWL ontologies. By default, it comes with 5.0.0 version of Protégé and the new Cellfie version is automatically updated with Protégé. To transfer data to ontology with Cellfie, open the previously prepared ontology in Protégé, click "Create axiom from Excel workbook…" and select the excel worksheet from "Open File" window. The incoming Cellfie window consists of five main components: "Sheet tab, Worksheet view, Transform rule edit panel, Transformation browser, and Create Axiom". To create the conversion rules, the "Add" button is selected in the conversion rule edit panel, and then an editor dialog opens where the conversion expression can be written. To import new axioms, transformation rules are prepared and continued by selecting the "Create Axioms" button. As seen in Fig. 11, after clicking the “Create Axiom” button, Cellfie automatically generated 10,025 OWL axioms and showed a preview of all of them. These new axioms, created with the help of the Cellfie plugin, can be imported into a new OWL as well as an existing open OWL.

**Fig. 12:**
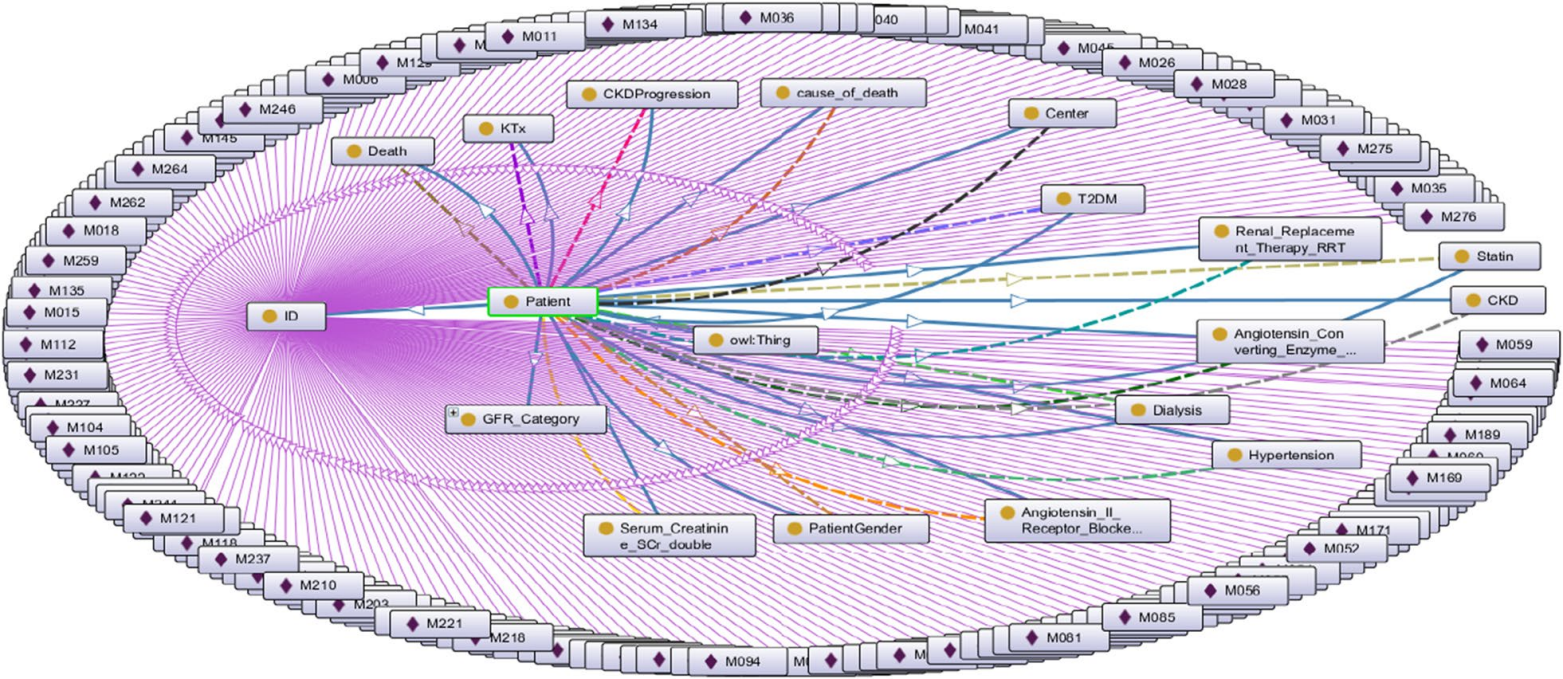
The OntoGraf of DIAKIDPatient Ontology

We use the Dataset [7] in excel format, which contains data on T2DM and CKD about 250 patients. In order to semantically annotate these patient data [73], we created the Patient ontology. The patients are members (instance) of the Patient class. In addition, the Patient class has several sub-classes such as Dialysis, Patient Gender, etc. To annotate information in the dataset, we also created necessary datatype and object properties (i.e. GFR, SCr, HbA1c, BMI, etc.) of CKD and T2DM patients in order to develop DIAKID for the treatment of these two diseases. Since CKD and T2DM diseases can cause many long and/or short-term complications in humans, the 'CKD Treatment' class has been added to our proposed ontology and used with the DMTO ontology that we have reused. The characteristic features of 249 patients were transferred to the ontology and the OntoGraf of DIAKIDPatient Ontology showing class, subclass, data properties, object properties, instances and relationships is presented in Fig. 12.

### Drug ontology

This chapter details the design of our proposed drug ontology and how it was developed. There are classes, subclasses, and individual characteristics of advanced drug ontology for both diseases (T2DM and CKD). Classes, subclasses and their individual characteristics are interrelated. For T2DM drugs, we re-used the DMTO ontology as we described in Sect. "Structure of the recommendation system". To our knowledge, there was no ontology for doses and interactions of CKD drugs critical to our research. Therefore, we created a new ontology of CKD drugs and drug doses as well as K-raising drugs using specialist physicians and KDIGO knowledge about CKD drug classes and doses [22, 74, 75]. And we also expanded its scope by adding K-raising drugs to our ontology. We expanded the scope of our study by transferring drug classes, drug active ingredients, drug starting doses and drug doses according to the GFR range to our ontology.

**Fig. 13:**
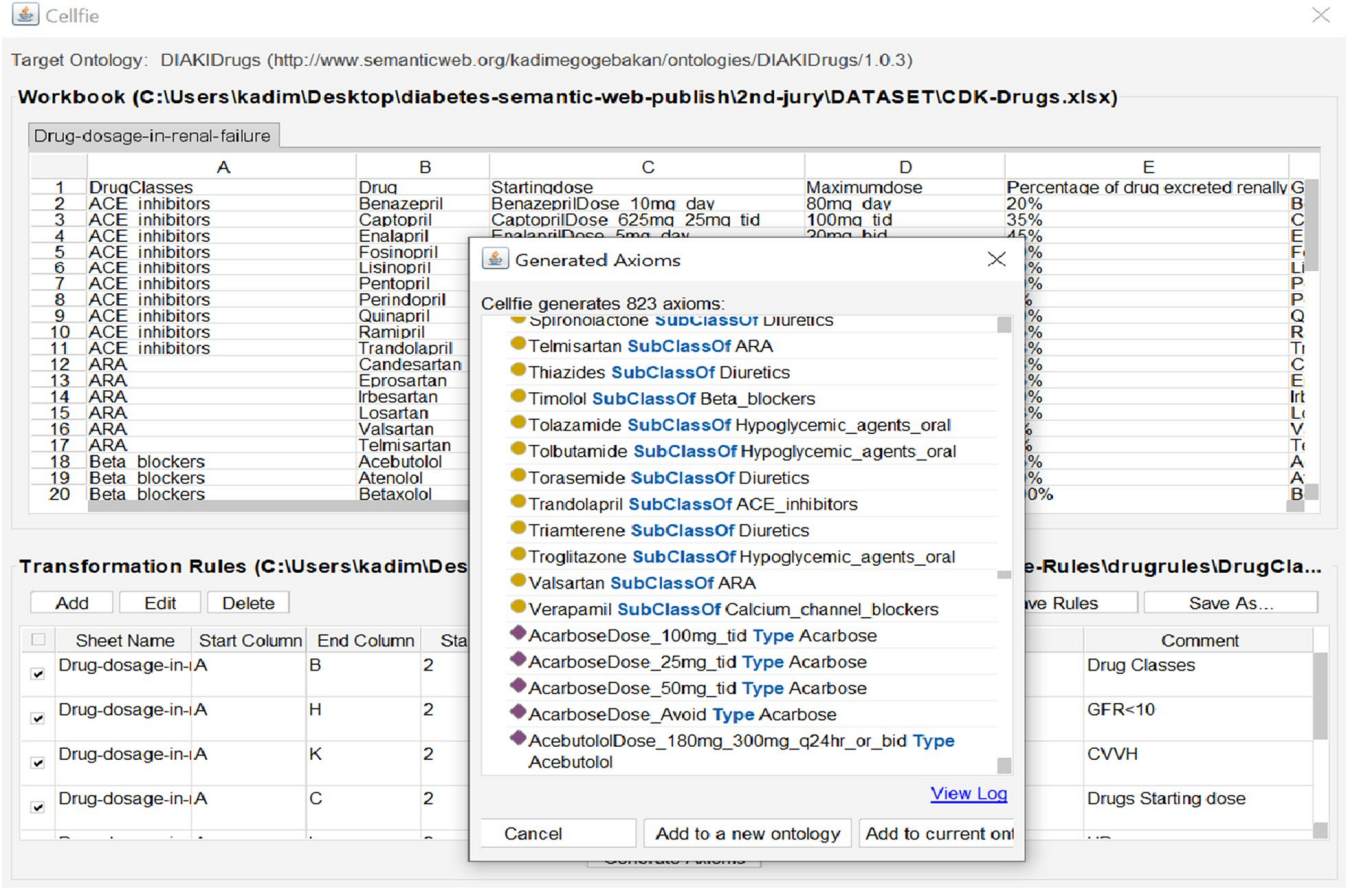
Use of Cellfie in the import of drug classes and subclasses with individuals

CKD drug classes consist of 7 main classes (Table 4). We used Cellfie to import the drug classes, subclasses and individual characteristics we received from the specialist physician to our ontology. Figure 13 shows the data import process to the Drug Ontology, the main classes, sub-classes and sample Drug individuals. In this process, a total of 823 axioms are created and transferred to our current ontology. The created Drug Ontology for CKD consists of 86 classes and 212 individuals.

**Table 4:** Information on drug doses according to eGFR in the treatment of CKD and T2DM [70]

Drug Class	Drug	Normal dosage		Dosage adjustment for renal failure with GFR (ml/min)		
		Starting dose	Maximum dose	> 50	10–50	< 10
ACE inhibitors	Benazepril	10 mg/day	80 mg/day	100%	75%	25%–50%
	Captopril	6.25–25 mg t.i.d	100 mg t.i.d	100%	75%	50%
	Enalapril	5 mg/day	20 mg b.i.d	100%	75%	50%
	–	–	–	–	–	–
ARA	Candesartan	16 mg/day	32 mg/day	100%	100%	50%
	Eprosartan	600 mg/day	400–800 mg/day	100%	100%	100%
	Irbesartan	150 mg/day	300 mg/day	100%	100%	100%
	–	–	–	–	–	–
Beta blockers	Atenolol	25 mg/day	100 mg/day	100%	75%	50%
	Betaxolol	20 mg q. 24 h	80–90%	100%	50%	50%
	Bopindolol	1 mg q. 24 h	4 mg q. 24 h	100%	100%	100%
	–	–	–	–	–	–
Calcium channel blockers	Amlodipine	2.5/day	10 mg/day	100%	100%	100%
	Diltiazem	30 mg t.i.d	90 mg t.i.d	100%	100%	100%
	Felodipine	5 mg b.i.d	20 mg/day	100%	100%	100%
	–	–	–	–	–	–
Diuretics	Acetazolamide	125 mg t.i.d	500 mg t.i.d	100%	50%	Avoid
	Amiloride	5 mg/day	10 mg/day	100%	100%	Avoid
	Bumetanide	1–2 mg/day	2–4 mg/day	100%	100%	100%
	–	–	–	–	–	–
Hypoglycemic agents (oral)	Acarbose	25 mg t.i.d	100 mg t.i.d	100%	50%	Avoid
	Chlorpropamide	100 mg q. 24 h	500 mg q24h	50%	Avoid	Avoid
	Glibornuride	12.5 mg q. 24 h	100 mg q. 14 h	No data	No data	No data
	–	–	–	–	–	–
Hypoglycemic agents (parenteral)	Insulin	Variable	Variable	100%	75%	50%
	Lispro insulin	Variable	Variable	100%	75%	50%

**Fig. 14:**
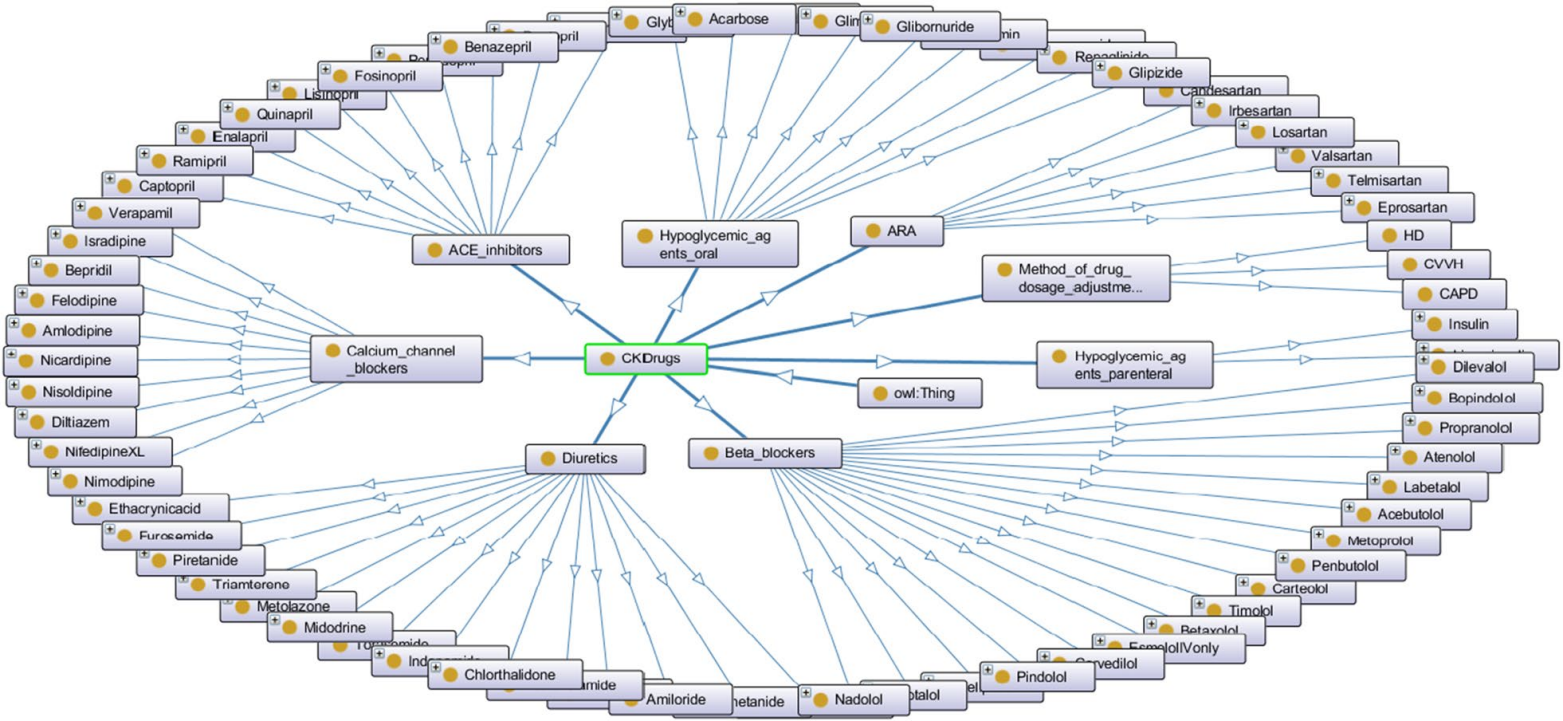
The OntoGraf of DIAKIDrugs Ontology

Table 4 includes drug classes, general names of drugs, maximum and minimum drug doses, and the change of drug doses according to the GFR value range of CKD. Not all drug lists for drug classes are presented in this table, they are abbreviated. CKD directly affects drug absorption, drug distribution, metabolism, and drug excretion [70]. In this disease, the patient's life can be endangered as a result of misuse of drug doses and may cause side effects by rendering the treatment ineffective. Doses of renally excreted drugs should be adjusted according to the patient's functioning level of kidney function, and renal function is calculated as the estimate GFR ratio [22]. The drug ontology was established according to KDIGO for Clinical Practice Guidelines for the Treatment of Diabetes in CKD. The OntoGraf of the DIAKIDrugs Ontology, consisting of classes, subclasses and individuals, are presented in Fig. 14 after all drugs (classes and subclasses) used in CKD Treatment are added to the Ontology along with their doses (individual).

### Drug-drug interaction ontology

**Fig. 15:**
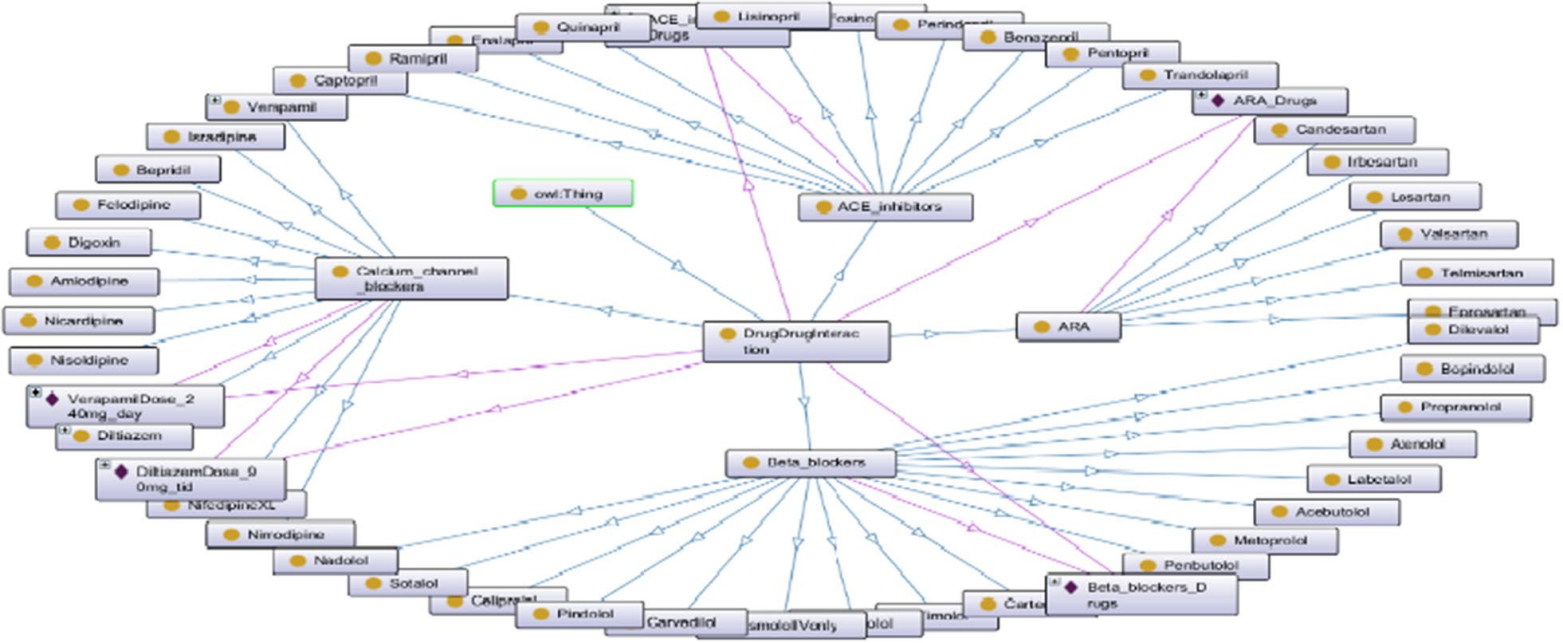
DDI Basic concepts and relationships

Again, DDI ontology, the knowledge of specialist physicians and KDIGO was used. Provided knowledge was used to create drug classes, subclasses and individual characteristics (instance), and the necessary information was transferred to the Protégé with the help of Cellfie. The DDI Ontology has been defined to warn of the interaction between drugs used for CKD disease and the outcome of this interaction. This work establishes the ontology of drug interactions, adopting the following steps: (1) Defines drug classes and drugs, (2) Analyses and states relationships between these drugs, and (3) creates rules of reasoning for drug interactions for these drugs. After all these stages, the basic concepts and relationships of the DDIOntology is created for DDI (Fig. 15).

**Fig. 16:**
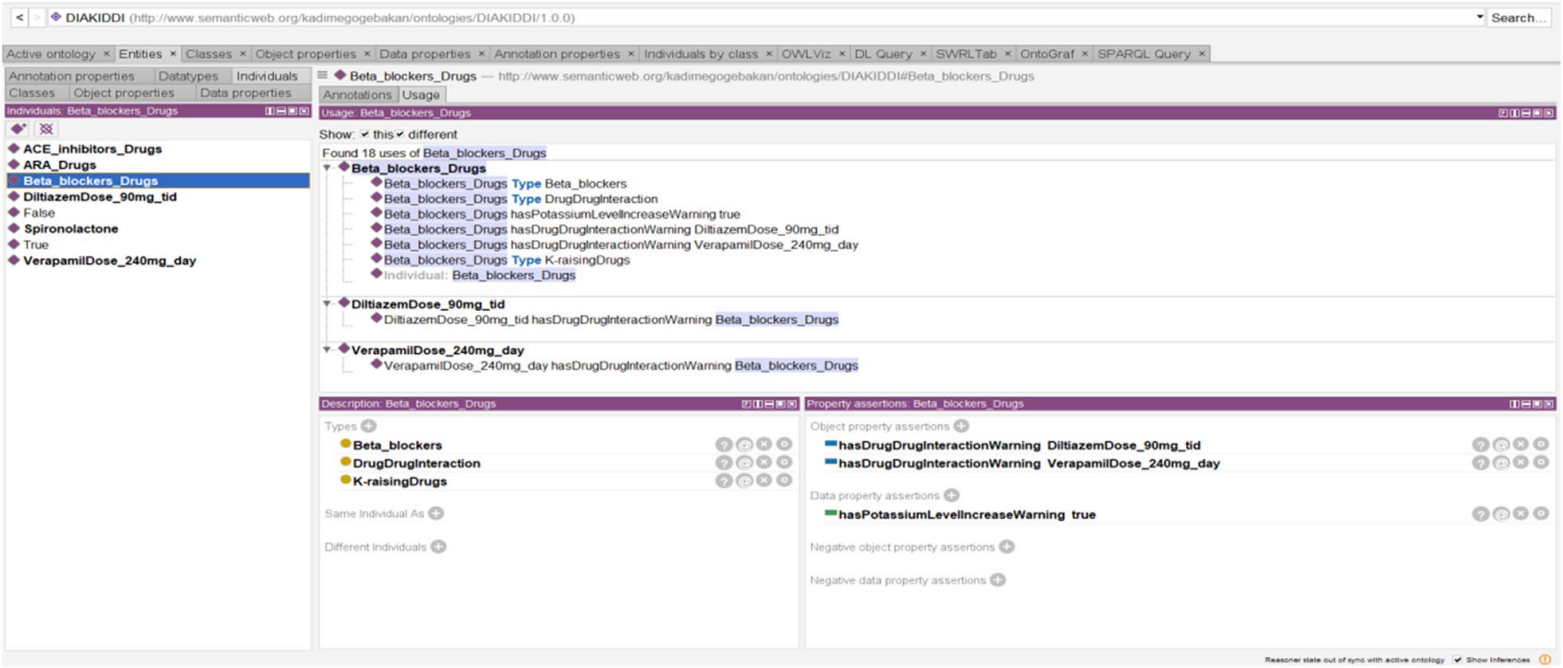
Drugs that increase potassium level and protégé interface

DDIs can cause side effects on the patient, and some drugs can increase the K level and put the patient in a difficult situation. Because the K level rises; Respiratory failure, respiratory irregularity, heart rhythm disorder and high amount of toxic substances in the heart can cause health problems that can seriously affect vital functions. As presented in Fig. 16, in order to draw attention to this situation, we added warnings about drugs that increase K levels, as well as DDI, to our system.

### DIAKID ontology

**Fig. 17:**
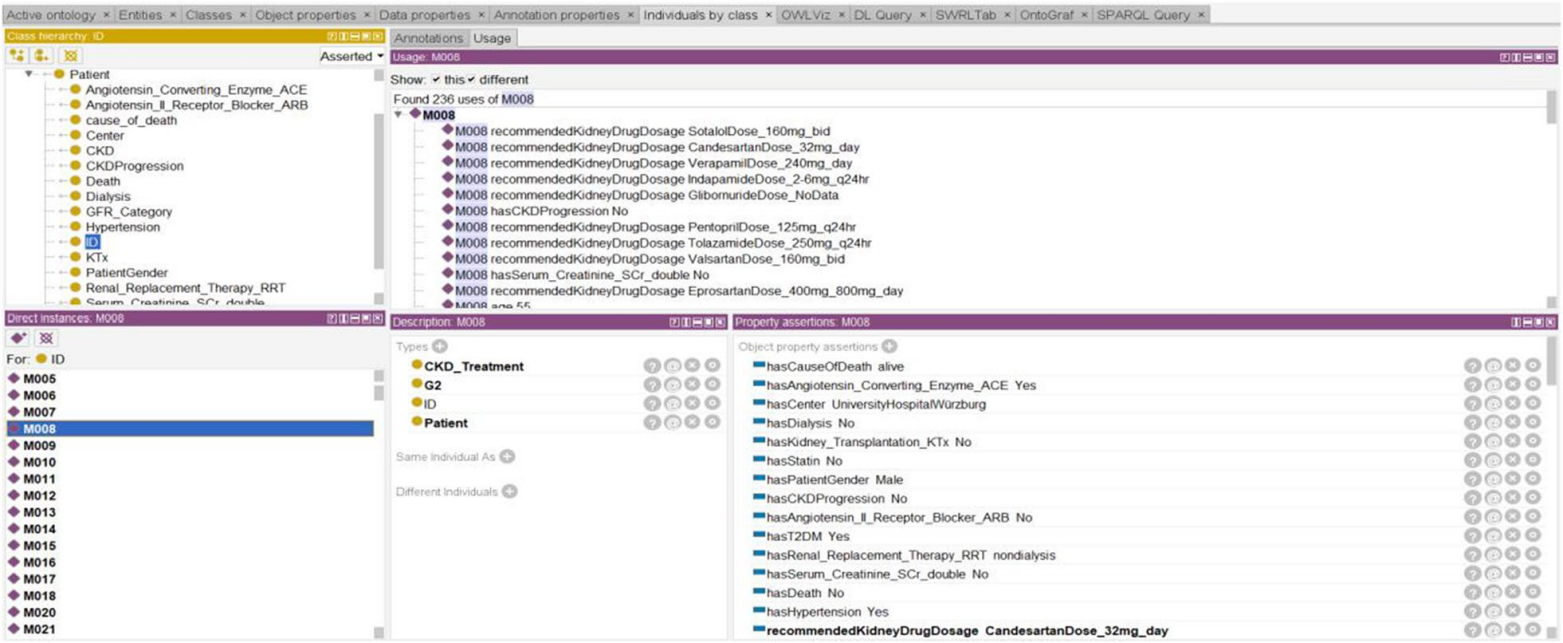
The developed DIAKID ontology in the protégé-OWL 5.5. showing patients and their dataset

**Fig. 18:**
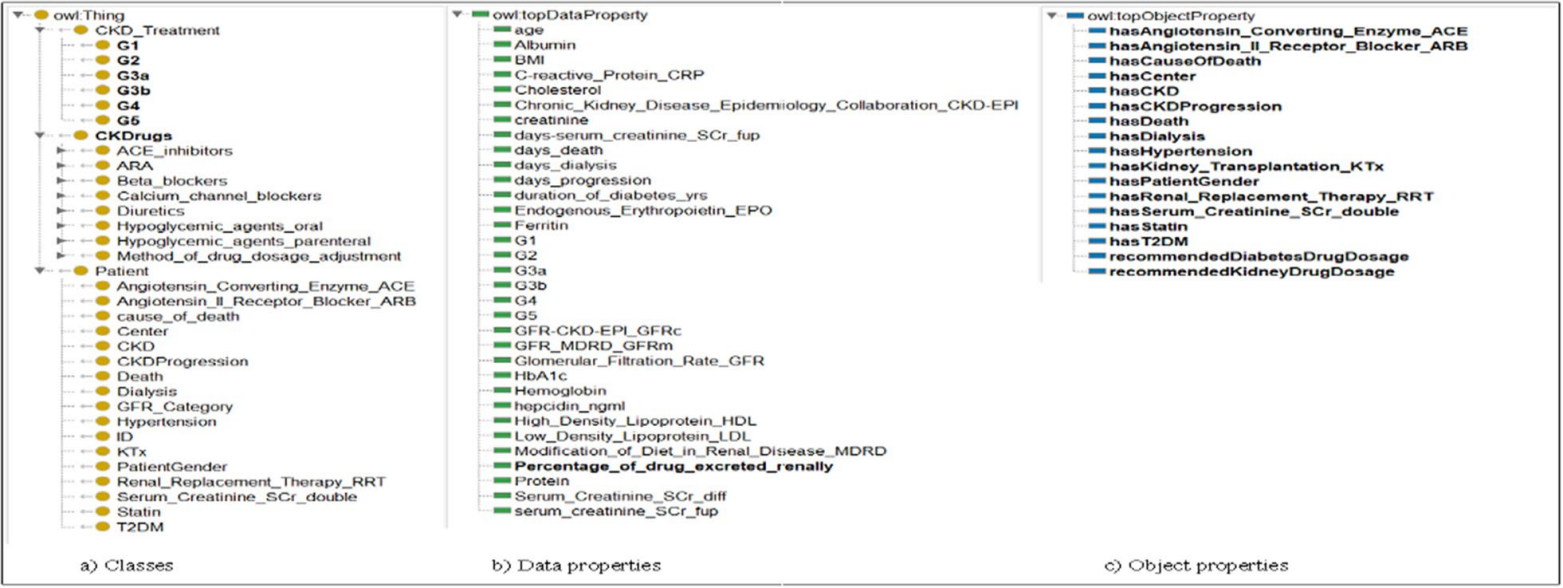
The developed new concepts in DIAKID? ontology in the protégé-OWL 5.5

**Fig. 19:**
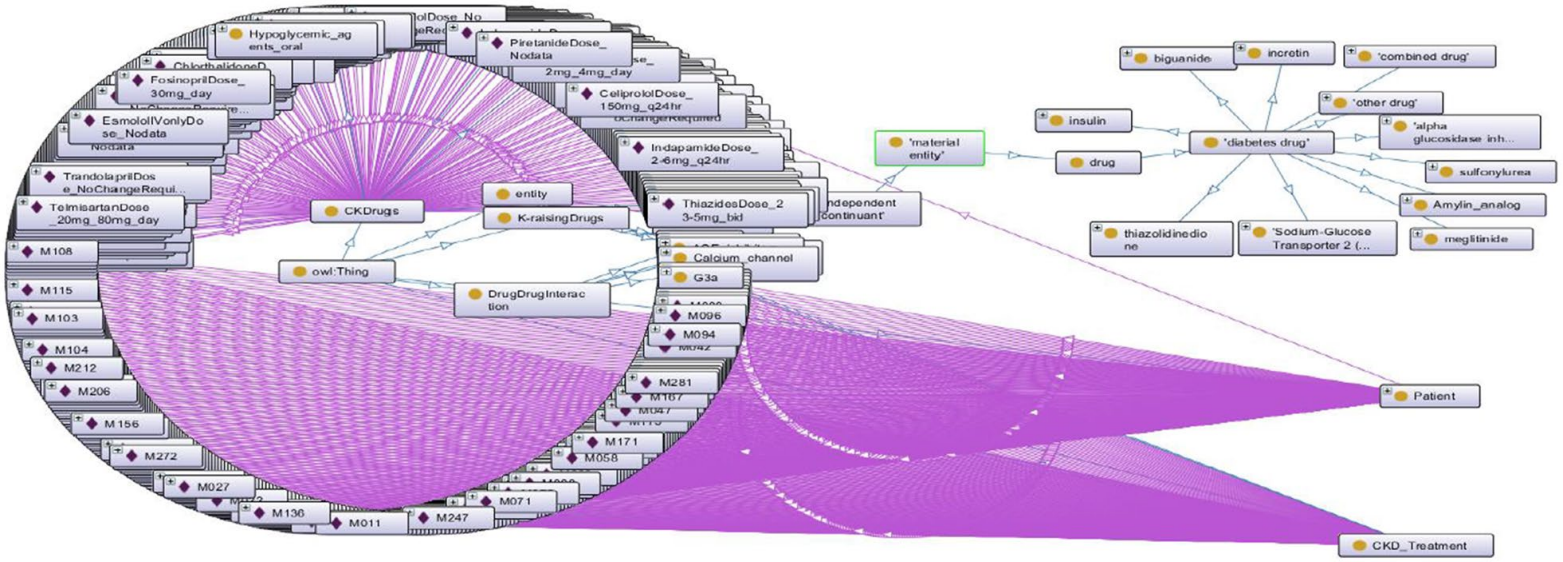
The OntoGraf of DIAKID Ontology

A detailed ontology was created to describe the concepts related to CKD disease/ treatment and the relationships between these concepts. The ontology layer defines concepts related to CKD stages and contains information about the doses of recommended drugs. To create classes of CKD Treatment, the KDIGO 2020 CPG and specialist physician knowledge were used. This ontological approach to staging the disease can more accurately predict the doses of drugs recommended for disease treatment based on data from the dataset for each patient. The patient's GFR category is required for the correct adjustment of drug doses in the management of CKD Patients. In this study, we overhaul the entire data set with SWRL rules and determine the GFR category of each patient. As a result of all re-used and newly created ontology, we form the novel DIAKID Ontology. Figure 17 shows stages of CKD and the doses of drugs recommended for the treatment of the disease for each patient using the proposed ontologies and SWRL rules. We will explain the DDI warning and drug dose recommendation process (Sect. "SWRL rules for drug-drug interaction and dose suggestion") using ontologies and SWRL rules in the coming sections. Figure 18(a) shows the generic classes for the DIAKID ontology and their subclasses. The generated classes have two kinds of properties, "data properties" to denote properties of particular class instances and "object properties" to describe relationships between different classes, as shown in Figs. 18(b, c), respectively. When we combined the newly created drug dose, drug-drug interaction and patient ontology with the DMTO ontology we modified, we obtained the DIAKID ontology, which is very dense with all classes, subclasses, data properties, object properties, individuals and relationships. The state of our ontology in question after all integrations is presented as OntoGrap in Fig. 19.

### Summary of ontologies used

**Fig. 20:**
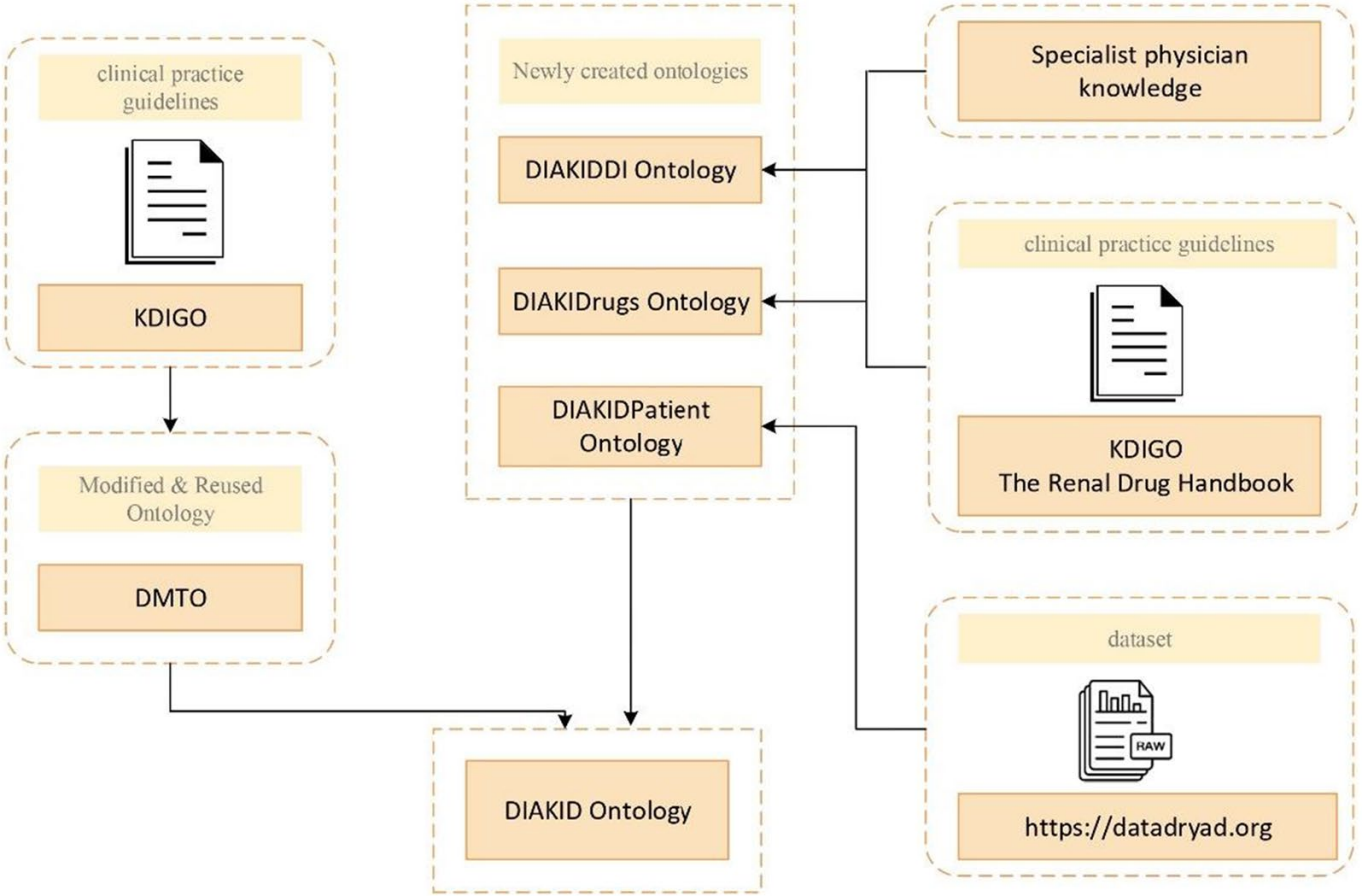
Structure of DIAKID Ontology

To develop our DIAKID System (Fig. 20), we combined the dataset (raw data) consisting of 41 characteristic features of 249 diabetic chronic kidney patients with three new and one modified ontology, using specialist physician knowledge and clinical practice guidelines data to form the proposed DIAKID ontology. In this section, we have presented the summary information about our DIAKID system, which we have created by integrating the new ontologies and the modified ontology. To improve our system, we developed three new ontologies ourselves and modified and reused one existing ontology. Statistical information about newly created ontologies and modified and reused ontology (Ontology Name, Class Subclass, Object Property, Data Property, and Individual) are given in Table 5.

**Table 5:** Statistics of reused and newly created ontologies

Ontology Name	Class	Subclass	Object Property	Data Property	Individual
modified DMTO	148	147	191	124	63
DIAKIDDI	44	46	1	1	37
DIAKIDDrugs	81	80	0	0	212
DIAKIDPatient	18	17	15	32	270
DIAKID	317	317	209	158	678

### Dataset and annotation with DIAKID

**Fig. 21:**
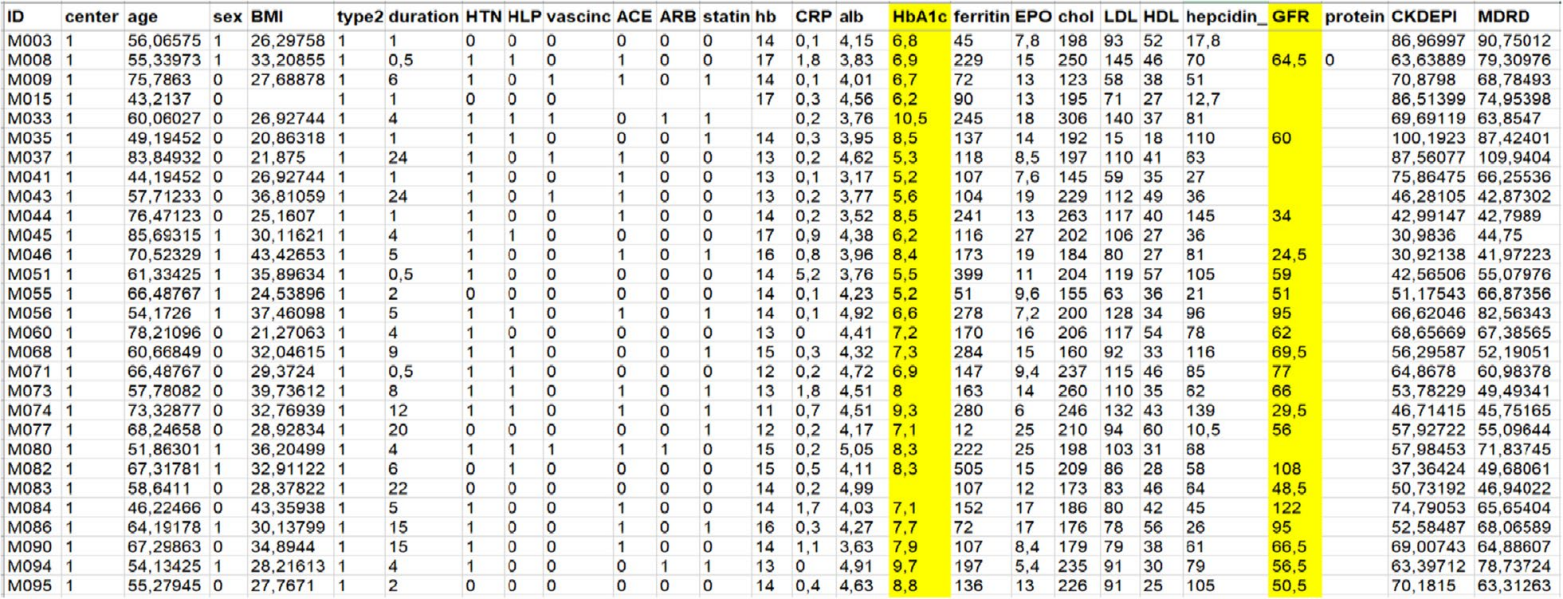
249 diabetic patients with and CKD at any stage

After doing research from many sources and web pages, we finally found a dataset that contains both T2DM and CKD patient data together and used it for our research. Our dataset, part of which is shown in Fig. 21, contains data from 249 diabetic patients with CKD at any stage, excluding ESRD (end-stage renal disease), who were followed for 4.2 years [7, 76]. The data we use to build our ontology will be available on request as we explain in Sect. "Methodology".

We use the proposed DIAKID ontology to annotate the dataset. A total of 41 rules were written to transfer patient data to the DIAKID RDF axioms. A total of 10,025 axioms were transferred to Protégé with the help of Cellfie.

### SWRL rules for drug-drug interaction and dose suggestion

While many drugs can have side effects on the patient, there are drugs that can cause side effects when used together. In this part of our study, we focused on DDIs that may cause adverse effects on the patient and adversely affect the patient's health if used together by diabetes patients with CKD. Diabetic patients with CKD cannot use ACE inhibitors and ARA drugs together [77]. Diltiazem and Verapamil from the Calcium Channel Blockers drug group cannot be used together with Beta blockers [78].

**Fig. 22:**
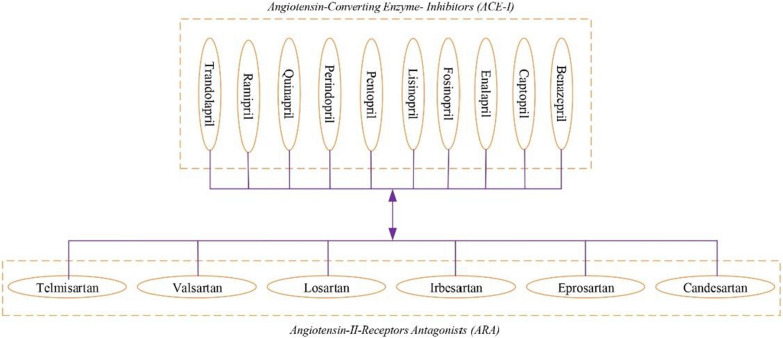
ACE-i and ARA Drug Lists, which drug-drug interacting when used together

In our system, rules have been added to the system to provide the necessary warnings about drugs as shown in Fig. 22; for example, any drug in the ACE inhibitors drug group will have a negative effect on the patient when used together with any drug in the ARA drug group.

**Fig. 23:**
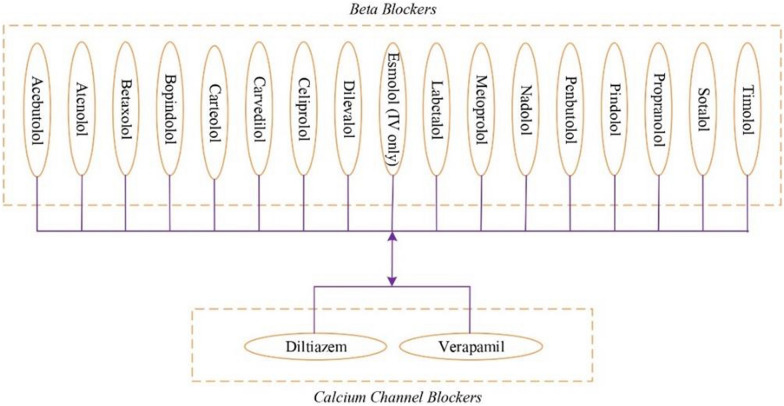
Beta Blockers and Calcium Channel Blockers Drug Lists, which DDI when used together

In our system, rules have been added to the system to provide the necessary warnings about all the drugs in Fig. 23, which explains that any drug in the Beta Blockers drug group will have a negative effect on the patient when used together with Diltiazem or Verapamil in the Calcium Channel Blockers drug group. Table 6 illustrates a DDI SWRL rule that we added to our system; when Benazepril in the ACE-i group is prescribed together with any drug in the ARA group, our system makes the necessary warnings to specialist physicians. We created SWRL rules to predict and warn of DDIs. The system will warn when any of the Beta Blockers drug group is prescribed together with Verapamil. Likewise, the system will warn again when any of the Beta Blockers drug group is prescribed together with Diltiazem. However, all drugs of the Beta Blocker group can interact with Verapamil and Diltiazem. As we explain in the SWRL rule in Table 7 below, the system makes the necessary warnings to specialist physicians when the drug Diltiazem, which is in the Calcium Channel Blockers drug group, is prescribed together with any of the drugs in the Beta Blockers drug group. A total of 34 SWRL rules have been created for DDI and adverse event warnings.

**Table 6:** Examples of DDI rules

Name	Rules
Rule:	p0:Candesartan(?x)—> p0:hasDrugDrugInteractionWarning(?x, p0:Benazepril) ^ p0:hasDrugDrugInteractionWarning(?x, p0:Captopril) ^ p0:hasDrugDrugInteractionWarning(?x, p0:Enalapril) ^ p0:hasDrugDrugInteractionWarning(?x, p0:Fosinopril) ^ p0:hasDrugDrugInteractionWarning(?x, p0:Lisinopril) ^ p0:hasDrugDrugInteractionWarning(?x, p0:Pentopril) ^ p0:hasDrugDrugInteractionWarning(?x, p0:Perindopril) ^ p0:hasDrugDrugInteractionWarning(?x, p0:Quinapril) ^ p0:hasDrugDrugInteractionWarning(?x, p0:Ramipril) ^ p0:hasDrugDrugInteractionWarning(?x, p0:Trandolapril)

**Table 7:** Examples of DDI rules

Name	Rules
Rule:	p0:Diltiazem(?x)—> p0:hasDrugDrugInteractionWarning(?x, p0:Acebutolol) ^ p0:hasDrugDrugInteractionWarning(?x, p0:Atenolol) ^ p0:hasDrugDrugInteractionWarning(?x, p0:Betaxolol) ^ p0:hasDrugDrugInteractionWarning(?x, p0:Bopindolol) ^ p0:hasDrugDrugInteractionWarning(?x, p0:Carteolol) ^ p0:hasDrugDrugInteractionWarning(?x, p0:Carvedilol) ^ p0:hasDrugDrugInteractionWarning(?x, p0:Celiprolol) ^ p0:hasDrugDrugInteractionWarning(?x, p0:Dilevalol) ^ p0:hasDrugDrugInteractionWarning(?x, p0:Labetalol) ^ p0:hasDrugDrugInteractionWarning(?x, p0:Metoprolol) ^ p0:hasDrugDrugInteractionWarning(?x, p0:Nadolol) ^ p0:hasDrugDrugInteractionWarning(?x, p0:Penbutolol) ^ p0:hasDrugDrugInteractionWarning(?x, p0:Pindolol) ^ p0:hasDrugDrugInteractionWarning(?x, p0:Propranolol) ^ p0:hasDrugDrugInteractionWarning(?x, p0:Sotalol) ^ p0:hasDrugDrugInteractionWarning(?x, p0:EsmololIVonly)

**Fig. 24:**
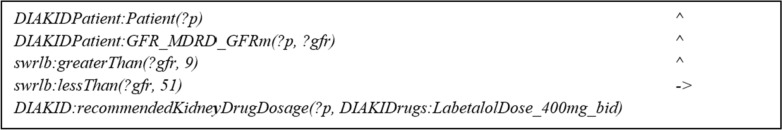
A simple SWRL rule

SWRL is an ontology-based rules language and was used to create rules. Drug regulation guidelines are from KDIGO. As shown in Fig. 24, the rules can be explained simply as follows: If the GFR level is < 51 and > 9, which dose and how can the drug be used for CKD according to the GFR and albuminuria category? Side effects of the drug, if the patient has been on dialysis, will the dosages change and what will be the things to watch? The following SWRL rule may conclude that a patient with a GFR between < 51 and > 9 can use Labetalol at 400 mg twice daily. The Knowledge Management System was used as editors to create and update the ontology model. The Rule Editor was used to edit and run the SWRL rules of the ontology model. The graphical communication interface enables users to operate the system easily and get the desired result. We created 325 SWRL rules to improve the proposed DIAKID ontology, providing warnings for DDI and recommending drug doses. Rules were created to give warnings for DDIs, drug doses, CKD stages of the patient, and K levels as follows.

### CKD Patient stage rules

According to the feedback we receive from specialist practitioners, GFR value range of the patient determines the drug doses used in the medication of T2DM and CKD. Therefore, 6 SWRL rules were created by looking at the GFR value of each patient to determine the CKD stage of each patient according to the GFR values given in Table 8. Two example rules are described in Table 8. In rule 1, if the patient's GFR is greater than 89, the patient has CKD in Stage G1. As stated in Rule 2 in the same table; If the patient's GFR test result is greater than 59 and less than 90, CKD is in Stage G2.

**Table 8:** The GFR stage of CKD patients

Name	Rule
Rule1:	DIAKIDPatient:Patient(?p) ^ DIAKIDPatient:GFR(?p, ?gfr) ^ swrlb:greaterThan(?gfr, 89)—> DIAKID:G1(?p)
Rule2:	DIAKIDPatient:Patient(?p) ^ DIAKIDPatient:GFR(?p, ?gfr) ^ swrlb:greaterThan(?gfr, 59) ^ swrlb:lessThan(?gfr, 90)—> DIAKID:G2(?p)

### DDI rules

Diabetic patients with CKD cannot use ACE inhibitors and ARA drugs together, and they cannot also use Calcium channel blockers Diltiazem and Verapamil with Beta blockers [3, 13]. 34 Rules were created using SWRL. In Rule1 in Table 9, the system will give a "hasDrugDrugInteractionWarning" warning when Acebutolol is prescribed with any of the drugs Diltiazem and Verapamil. As seen in Rule 2 in the same table; When Verapamil is prescribed together with any drug from the Beta blocker drug group (Acebutolol, Atenolol, Betaxolol, …), the system will warn "hasDrugDrugInteractionWarning".

**Table 9:** DDI warnings written with SWRL rules

Name	Rule
Rule1:	Acebutolol(?x)—> hasDrugDrugInteractionWarning(?x, Diltiazem) ^ hasDrugDrugInteractionWarning(?x, Verapamil)
Rule2:	Verapamil(?x)—> hasDrugDrugInteractionWarning(?x, Acebutolol) ^ hasDrugDrugInteractionWarning(?x, Atenolol) ^ hasDrugDrugInteractionWarning(?x, Betaxolol) ^ hasDrugDrugInteractionWarning(?x, Bopindolol) ^ hasDrugDrugInteractionWarning(?x, Carteolol) ^ hasDrugDrugInteractionWarning(?x, Carvedilol) ^ hasDrugDrugInteractionWarning(?x, Celiprolol) ^ hasDrugDrugInteractionWarning(?x, Dilevalol) ^ hasDrugDrugInteractionWarning(?x, Labetalol) ^ hasDrugDrugInteractionWarning(?x, Metoprolol) ^ hasDrugDrugInteractionWarning(?x, Nadolol) ^ hasDrugDrugInteractionWarning(?x, Penbutolol) ^ hasDrugDrugInteractionWarning(?x, Pindolol) ^ hasDrugDrugInteractionWarning(?x, Propranolol) ^ hasDrugDrugInteractionWarning(?x, Sotalol) ^ hasDrugDrugInteractionWarning(?x, EsmololIVonly)

### Drug dosage rules

As a result of the values, we obtained from the dataset [77], we wrote 276 rules by looking at GFR of each diabetic patient with CKD and recommended the drug dose accordingly. Two of these rules are shown in Table 10. In Rule 1, if the patient is a Chronic Kidney Patient, not a Dialysis patient and has a GFR between 9 and 51; The dose to be used for the drug Acarbose is 50 mg tid (three times a day). In rule 2, if the patient is not a Dialysis patient, but has a CKD and GFR is less than 10; Acebutolol dosage is 180 mg or 300 mg once a day.

**Table 10:** Warnings about drug doses written in SWRL rules

Name	Rule
Rule1:	DIAKIDPatient:Patient(?p) ^ DIAKIDPatient:hasCKD(?p, DIAKIDPatient:Yes) ^ DIAKIDPatient:hasDialysis(?p, DIAKIDPatient:No) ^ DIAKIDPatient:GFR(?p, ?gfr) ^ swrlb:greaterThan(?gfr, 9) ^ swrlb:lessThan(?gfr, 51)—> DIAKID:recommendedKidneyDrugDosage(?p, DIAKIDrugs:AcarboseDose_50mg_tid)
Rule2:	DIAKIDPatient:Patient(?p) ^ DIAKIDPatient:hasCKD(?p, DIAKIDPatient:Yes) ^ DIAKIDPatient:hasDialysis(?p, DIAKIDPatient:No) ^ DIAKIDPatient:GFR(?p, ?gfr) ^ swrlb:lessThan(?gfr, 10)—> DIAKID:recommendedKidneyDrugDosage(?p, DIAKIDrugs:AcebutololDose_180mg_300mg_q24hr_or_bid)

### K-raising drug rules

4 Rules were created using SWRL for 34 drugs in 4 different drug classes. Below are a few rules we wrote about ACE-i, ARA, Beta Blocker drug groups and the ability of Spironolactone to increase K levels. In Rule2 in Table 11; When any of the drugs Benazepril, Captopril, Enalapril, Fosinopril, Lisinopril, Pentopril, Perindopril, Quinapril, Ramipril and Trandolapril are prescribed, the system will give a "hasPotassiumLevelIncreaseWarning(?a, true)" warning.

**Table 11:** SWRL rule warning for drugs that raise potassium levels

Name	Rule
Rule1:	Spironolactone(?a)—> hasPotassiumLevelIncreaseWarning(?a, true)
Rule2:	DIAKIDrugs:Benazepril(?a) ^ DIAKIDrugs:Captopril(?a) ^ DIAKIDrugs:Enalapril(?a) ^ DIAKIDrugs:Fosinopril(?a) ^ p0:Lisinopril(?a) ^ DIAKIDrugs:Pentopril(?a) ^ DIAKIDrugs:Perindopril(?a) ^ p0:Quinapril(?a) ^ DIAKIDrugs:Ramipril(?a) ^ DIAKIDrugs:Trandolapril(?a)—> p0:hasPotassiumLevelIncreaseWarning(?a, true)

### Rules for T2DM drug dose

Here are some examples of rules we wrote for classes, data property and object property that we imported from DMTO to our own ontology. In Rule1 in Table 12, if the patient has diabetes and his GFR is greater than 29; For lispro_mix dose usage the system will result in "Reduce_Dose_by_25_percent".

**Table 12:** SWRL rules for T2DM drug doses

Name	Rule
Rule1:	DIAKIDPatient:Patient(?p) ^ DIAKIDPatient:hasT2DM(?p, DIAKIDPatient:Yes) ^ DIAKIDPatient:GFR(?p, ?gfr) ^ swrlb:greaterThan(?gfr, 9) ^ swrlb:lessThan(?gfr, 51)—> DIAKID:recommendedDiabetesDrugDosage(?p, autogen0:_lispro_mix-Reduce_Dose_by_25_percent)
Rule2:	DIAKIDPatient:Patient(?p) ^ DIAKIDPatient:hasT2DM(?p, DIAKIDPatient:Yes) ^ DIAKIDPatient:GFR(?p, ?gfr) ^ swrlb:lessThan(?gfr, 10)—> DIAKID:recommendedDiabetesDrugDosage(?p, DIAKID:Aspart_NovoRapid-Reduce_Dose_by_50_percent)

### Evaluation and results

#### Evaluation setup and metrics

**Fig. 25:**
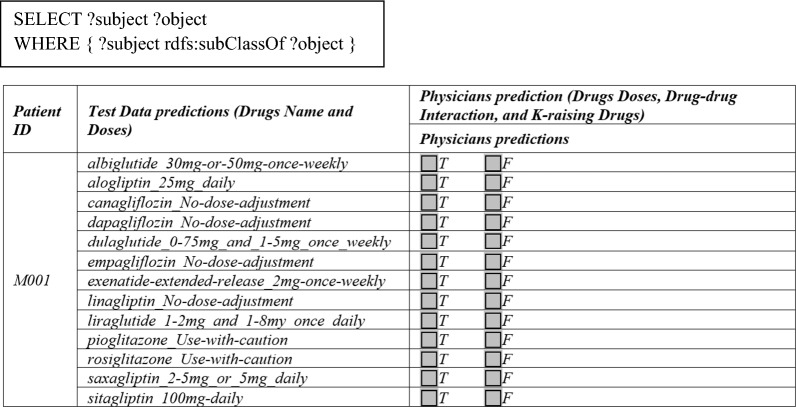
A sample ground truth data compare with test data for Patient M001

The end goal of the proposed system is to accurately predict (a) drug dose, (b) DDI, and (c) K-raising drug warnings for patients having both diabetes and CKD. As we discussed before, we use the publicly available dataset [22] that includes information about patients having both T2DM and CKD. Using the available patient data, our system predicted drug names, drug doses, possible DDI warnings and potassium level warnings for each patient. We used the dataset [22] for quantitative evaluations. After carefully investigating patient data, patients who passed away were removed from the user study. In total, we have 153 patients for the evaluations. Although the used dataset is publicly available, we need the opinions of specialist doctors on our predictions for quantitative analysis. First, we received ethics approval from the Near East University ethics committee to carry out user evaluations with three specialist doctors. For quantitative evaluations, we asked three specialist doctors to rate the predictions of the proposed system for drug names, drug doses, possible DDI warnings and potassium level warnings. We sent the drug lists recommended by our system for each patient to the physicians in Excel format for evaluation, as seen in Fig. 25. Physicians evaluated the system by stating their opinions as True (T) or False (F). The specialist physicians rate their opinions independently, therefore, they were not aware of the ratings of other specialists. Analysis of the results is performed against: (a) using individual ratings of each specialist (accepted as ground truth) and (b) agreed opinions of three specialists are accepted as ground truth (i.e. majority voting). Then, we compare the ground truth with the recommendations given by the proposed system (i.e. test data) using DIAKID ontology and extensive SWRL rules. In this way, the effectiveness of the proposed system can be assessed.

Datasets for evaluation by specialist physicians are created by retrieving the information collected in the ontology for each patient using the following SQL, then executing it and copying the result directly into an Excel sheet as shown in Fig. 25. For the assessment of the performance, “Accuracy, Sensitivity, Specificity and Precision” quantitative metrics [79–83] are applied. The number of individuals correctly classified as True Positives (TPs), the number of non-drug individuals misclassified as drugs are False Negatives (FNs), the number of individuals correctly classified as drugs are True Negatives (TNs), and the number of individuals misclassified as drugs are True Negatives (TNs). The number of non-medicated individuals are False Positives (FPs) [84]. The formula is shown in Eq. (1).

#### $Accuracy=\frac{TN+TP}{TN+TP+FN+FP}$

By comparing the performance of the proposed DIAKID with the drug doses prescribed by the specialist, we evaluated our model in terms of specificity and sensitivity in drug dose estimation. The true-negative ratio is the specificity that measures the proportion of correctly classified TNs. Equation (2) is the calculation method of specificity.

#### $Specificity=\frac{TN}{TN+FP}$

Sensitivity is a method of calculating how well we define the true-positive ratio as recommending the correct drug in our system-identified TPs. The formula of the sensitivity is shown in Eq. (3) [85].

#### $Sensitivity=\frac{TP}{TP+FN}$

Precision calculated the ratio of TPs to the sum of TPs and FPs measurement.

#### $Precision =\frac{TP}{TP+FP}$

Mean Squared Error (MSE) measures the mean squared error rate of predicted values (P) against the actual/ground truth values (A).

5 $$MSE=\frac{1}{n}\sum_{i=1}^{n}{({A}_{i}-{P}_{i})}^{2}$$

#### Results and discussions of accuracy, sensitivity and specificity evaluations

The proposed system predicts; (1) drug doses for patients having both T2DM and CKD, (2) DDI warnings and (3) drugs that increase potassium (K) according to the patient data [7, 76] and extensive SWRL rules. System prediction results are converted from ontology to Excel format as explained above. Then, the excel data is transmitted to three specialist physicians for the evaluation of the system.

#### Performance analysis of DIAKID

Accuracy, Sensitivity and Specificity results are shown in Table 13, 14 and 15 respectively. The results show the performance of the proposed system predictions as opposed to the accepted ground truth data of each expert (i.e., Doctors I, II, and III) and based on their agreed opinions (i.e., majority vote). First, the accuracy of predictions is calculated based on individual specialist voting. Then, the agreed votes of three specialists are compared. Table 13 shows that the proposed system achieves an average accuracy of 95%-97% when ground truth data of individual physicians are considered. When majority voting is applied based on the agreed ground truth of three physicians, the proposed DIAKID system achieves an accuracy of 96%, 97% and 97% in terms of drug dose estimation, DDI estimation, and K-raising drug estimation respectively.The results are very promising.

**Table 13:** All Ground Truth Data for Accuracy

Ground Truth Data form	Accuracy for		
	Drug Dose	DDI	K-raising Drugs
Physician I	95%	97%	97%
Physician II	97%	97%	97%
Physician III	97%	97%	97%
Agreed	96%	97%	97%

**Table 14:** All Ground Truth Data for Sensitivity

Ground Truth Data form	Sensitivity for		
	Drug Dose	DDI	K-raising Drugs
Physician I	98%	98%	97%
Physician II	98%	98%	97%
Physician III	98%	98%	97%
Agreed	98%	98%	97%

**Table 15:** All Ground Truth Data for Specificity

Ground Truth Data form	Specificity for		
	Drug Dose	DDI	K-raising Drugs
Physician I	94%	96%	98%
Physician II	97%	96%	98%
Physician III	97%	96%	98%
Agreed	96%	96%	98%

Tables 14 and 15 show are observed for the sensitivity and specificity metrics. Results in Table 14 shows that the proposed DIAKID system has an agreed sensitivity rate of 98%, 98% and 97% for drug dose estimation, DDI estimation, and K-raising drug estimation respectively. The sensitivity of drug dose and DDI (i.e. 98%) is higher than the accuracy metric (i.e. 97%). The proposed system achieves an agreed specificity rate of 96%, 96% and 98% for drug dose estimation, DDI estimation, and K-raising drug estimation respectively. It appears that the specificity of K-raising drug estimation (98%) is higher than drug dose estimation and DDI estimations.

**Fig. 26:**
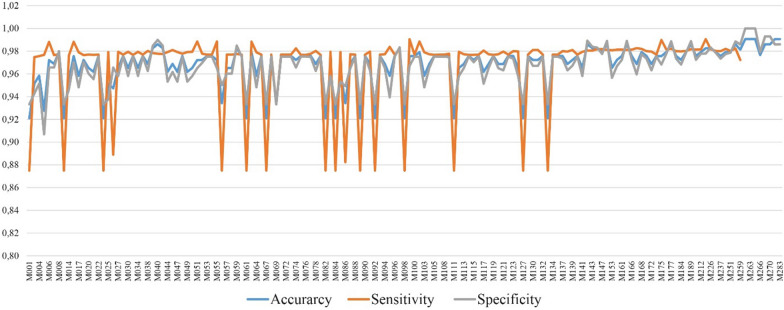
Comparison of accuracy, specificity, and sensitivity metrics for all patients

Overall, it can be said that the proposed DIAKID performs well in terms of sensitivity, specificity and accuracy. To better illustrate how the proposed system performs against each patient, accuracy, sensitivity and specificity graph for each individual patient is presented in Fig. 26. Looking at the results in Fig. 26, the Sensitivity of 15 patients appears to be lower than the others. When we examined these 15 patients, we observed that all of them had hasCDK: No, hasDialysis: No, hasT2DM: Yes. Although the number of drugs that can be used for this patient group is high, the number of drugs recommended by our system remains limited, and therefore, it reduced the sensitivity.

Drug dose prediction results in Table 13, 14 and 15 include the prediction results of both T2DM and CKD drug doses. In order to analyse how well the proposed system predicts diabetes and kidney diseases drug doses separately for patients, we separate the results into two: T2DM drug predictions and CKD drugs predictions as shown in Table 16. Results show that accuracy and sensitivity are more reliable for recommending CKD drugs than T2DM drugs. Specificity is higher for T2DM drug recommendation compared to recommending CKD drugs.

**Table 16:** Evaluation of drug dose predictions of DIAKID system for T2DM and CKD drugs separately

Metrics	T2DM	CKD
Accuracy	0,95	0,96
Sensitivity	0,96	0,98
Specificity	0,95	0,93

**Fig. 27:**
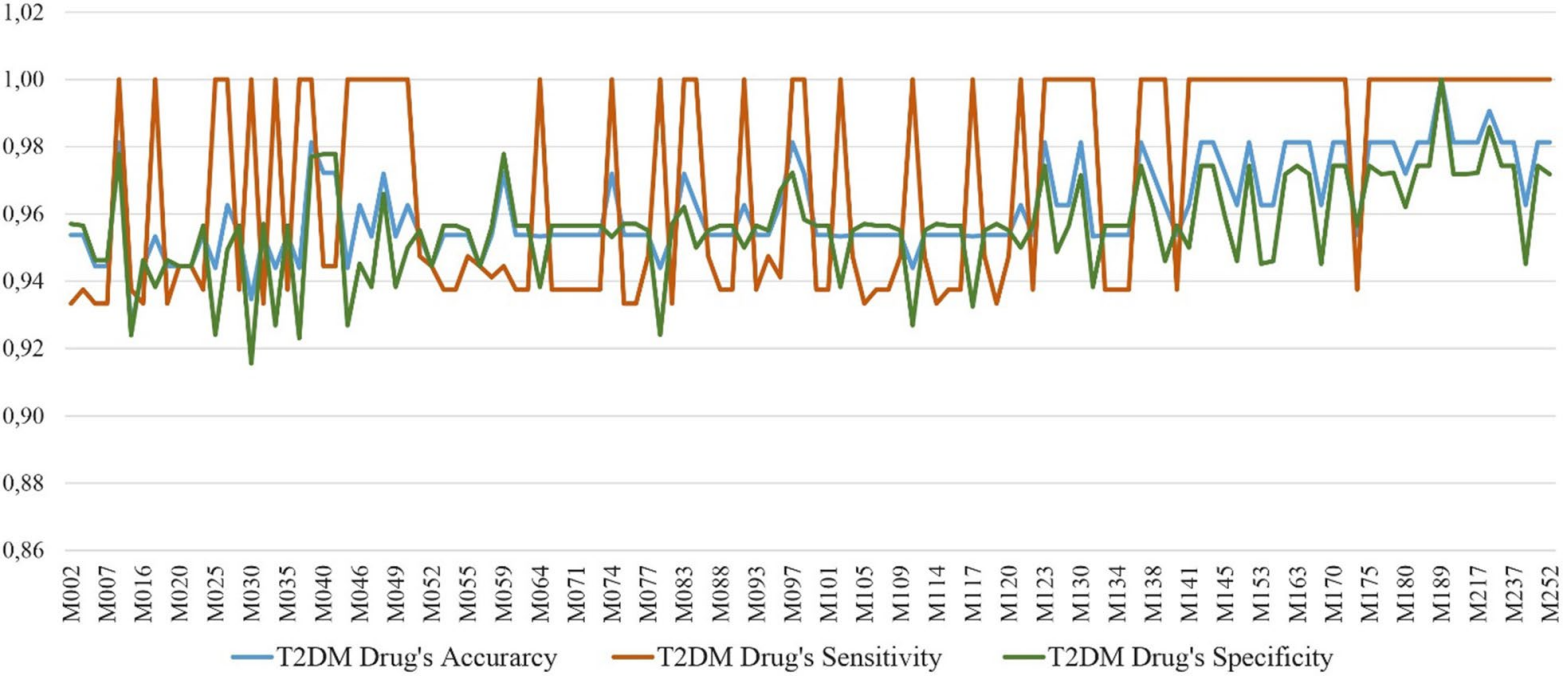
T2DM drug dose prediction results of the DIAKID system for each patient (Accuracy, Sensitivity and Specificity):

**Fig. 28:**
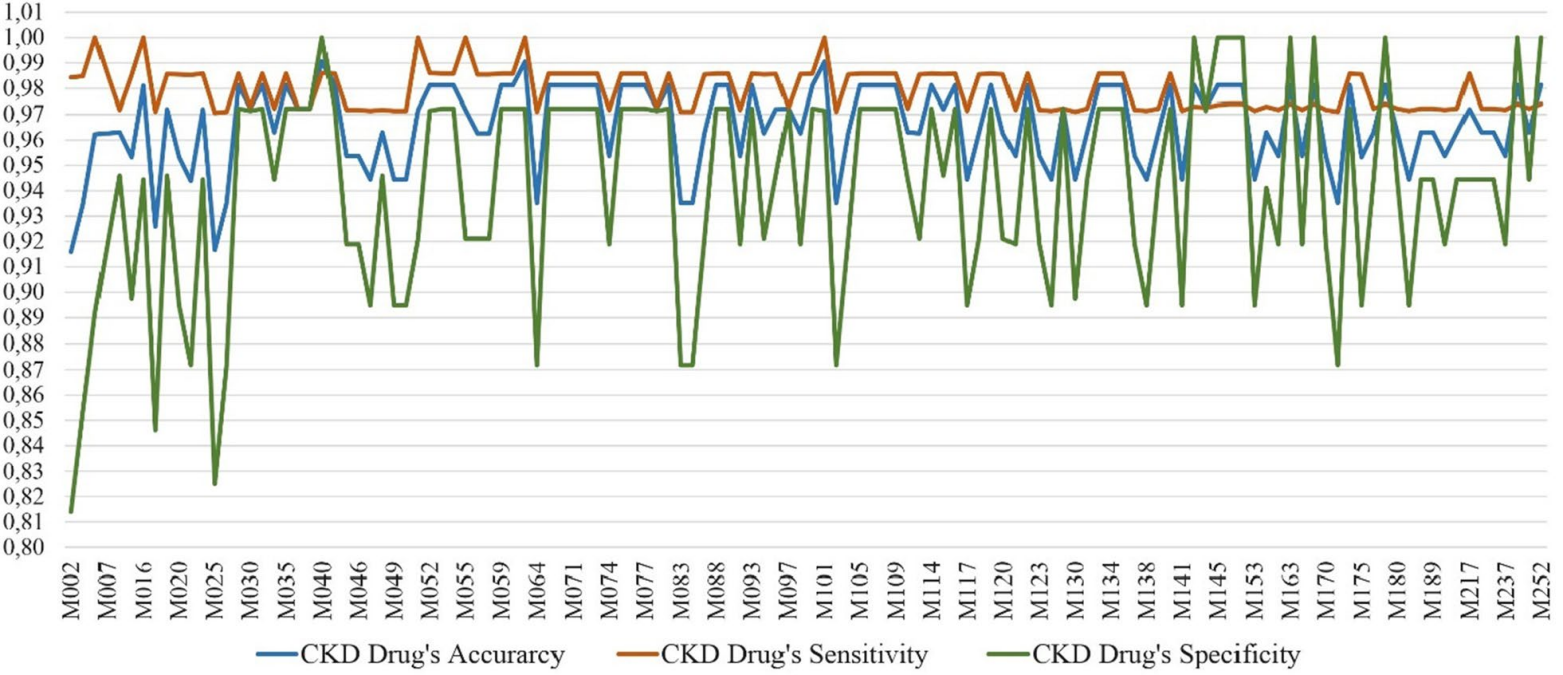
CKDM drug dose prediction results of the DIAKID system for each patient (Accuracy, Sensitivity and Specificity)

**Fig. 29:**
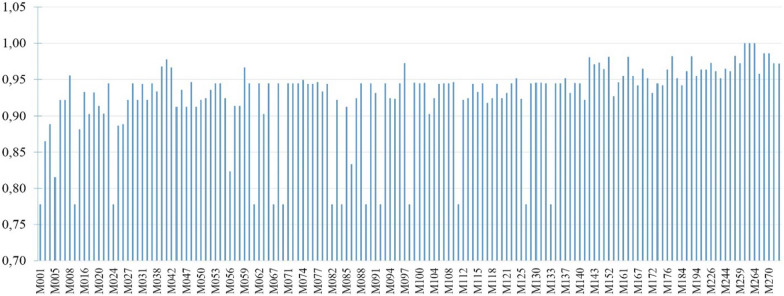
Precision results for each patient

Furthermore, in Figs. 27 and 28, T2DM and CKD drug dose predictions of the proposed system is illustrated for each patient in terms of accuracy, sensitivity and specificity. Results illustrate that CKD sensitivity metric is more stable than the sensitivity metric of T2DM for patients; meaning CKD drug doses are predicted better. On the other hand, CKD specificity metric for patients is lower than T2DM specificity metric which also explains the decreased specificity rate of 93% of CKD compared to 95% of T2DM (Table 16). The precision of drug dose predictions for each patient is also presented in Fig. 29. The average precision rate of our system is found to be 92%. Results in Fig. 29 also demonstrate that expect few cases, generally drug dose predictions are above 90%.

**Fig. 30:**
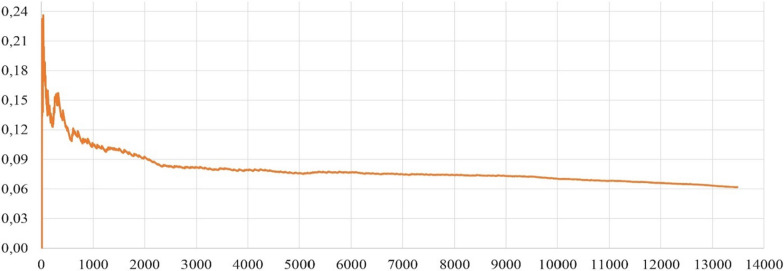
Mean Squared Error of drug doses for patients

Finally, for drug doses prediction, MSE graphic can illustrate the mean squared error for increasing number of patients in our dataset. The results show that the MSE value gradually decreases and the overall MSE value is 0.06. Because there are very few errors regarding the drug doses suggested by our system (Fig. 30).

#### Comparison with related work

Finally, we compared the performance of our proposed ontology with other ontologies as in Table 17. First, we illustrate individual results for drug dose, DDI and K-Raising drugs. Then, the performance of all predictions is combined by taking the average for drug dose, DDI and K-raising drugs for each metric. Compared with the related work, the aim of the proposed work is novel which is providing drug doses, DDI warnings and potassium level warnings for T2DM and CKD patients. In addition, when we compare the results of individual/average predictions for Drug dose, DDI and K-Raising drugs, we observe that the proposed work also achieves promising outcomes. Please note that existing works in Table 17 provide an ontology-based solution to a single disease such as diabetes. On the other hand, the proposed work takes two diseases (T2DM and CKD) into account while predicting correct drug dose amounts for T2DM and CKD, as well as, providing necessary warnings for DDIs and potassium levels. Even though we provide complex reasoning, accuracy, sensitivity and specificity metrics are still very high and competitive compared to the state-of-the-art methods (ranging 96–98%). When the drug dose recommended by our DIAKID System for 153 patients with only T2DM and CKD is evaluated using the Accuracy, Sensitivity and Specificity metrics, it can be said that it is more successful in recommending CKD drug doses. Overall, for the first time, the proposed system provides promising prediction results of drug doses, DDIs and K-raising drug warnings for patients having both T2DM and CKD.

**Table 17:** Summary of a comparative study between the DIAKID system and existing studies

Source	Accuracy	Sensitivity	Specificity	Aim
Zhang, Y. F. et al. 2017 [23]	99.93%	99.94%	99.88%	**Patient follow-up system evaluation:** Monitoring of T2DM patients from home by providing computerized clinical decision support
Chen, L. et al. 2019 [39]	98%	98.2%	99.2%	**Diabetes diagnosis:** Providing diabetes mellitus risk estimation, screening and treatment
Mallika, C. et al. 2019 [52]	81.2%	93.2%,	74.23% s	**Diagnosis of diabetes type**: Ensuring early diagnosis of diabetes and providing up-to-date information to physicists
DIAKID Ontology (Agreed)	96% (Drug Dose) 97% (DDI) 97% (K-Raising) 96.7% (avg.)	98% (Drug Dose) 98% (DDI) 97% (K-Raising) 97.7% (avg.)	96% (Drug Dose) 96% (DDI) 98% (K-Raising) 96.7% (avg.)	**Medication recommendation for T2DM and CKD patients:** Ensuring that warnings are issued for DDIs, drug doses, and drugs that raise K levels for multiple prescription patients

Bold emphasizes the aim of the compared works

#### Adapting the proposed work to other applications

The proposed method is not only limited to drug prescription warnings for T2DM and CKD diseases. It can be applied to other domains. "Heart Failure" and "Hypertension" are two good examples of these diseases [15]. In the treatment of "Heart Failure" and "Hypertension" diseases, which are complications of CKD, it is necessary to pay attention to drug dose adjustment and drug-drug interactions. Because the excretion or retention of drugs from the body depends entirely on the functioning of the kidney. Therefore, drug dosages used in the treatment of CKD and heart failure require adjustment [86]. Likewise, in the treatment of CKD and high blood pressure, the drug dosage must be adjusted to control blood pressure [87–89]. In conclusion, if treatment for another chronic disease is ongoing along with CKD, attention should be paid to drug dose adjustment, drug-drug interactions, and drugs that increase potassium levels. We can adapt the proposed system to these new domains by extending the active ingredients of drugs in DIAKID, as well as, extending the SWRL rules.

## Conclusions and future work

In our study, we developed an ontology-based assistive technology for the treatment of patients with both diabetes and chronic kidney disease and who were prescribed multiple prescriptions. In particular, we explained the proposed DIAKID Ontology that is consisting of modified DMTO ontology and novel ontologies of drugs, drug-drug interactions and patient profile ontologies. Based on the patient data, we automatically predict the correct dose of T2DM drugs, CKD drugs, drug-drug interaction warnings and increase potassium level warnings using extensive semantic rules. To assess the effectiveness of the proposed ontology-based system, first an open-source dataset was used. The dataset was converted to RDF format using the proposed DIAKID ontology. Finally, for each patient, drug estimates, drug-drug interaction warnings and potassium level warnings were predicted based on 324 complex semantic rules. Experiments on 153 patients show that the proposed system can achieve an average of 97.7% sensitivity and 96.7% specificity 96.7% accuracy compared to agreed expert advice. Other detailed analyses of results also illustrate that the proposed system is promising for predicting drug doses for patients having both T2DM and CKD. Other related works generally take into account a single disease (i.e. diabetes), whereas the proposed work takes into account both diseases of T2DM and CKD.

In the future, we will expand the scope of our study and improve our ontology by adding the effects of the drugs used in the treatment of T2DM-related diseases and complications on patients. We plan to develop a user-friendly interface for clinicians in the future.

## Data Availability

Data will be available on request.
